# *β*‐Caryophyllene‐Rich *Mercurialis perennis* Leaf Essential Oil: GC–MS Profiling, Antioxidant Activity, Molecular Docking, and Molecular Dynamics Analysis

**DOI:** 10.1002/cbdv.71337

**Published:** 2026-05-15

**Authors:** Chika Attama, Jean Noël Nyemb, Lawrence Luka, Herve Landry Ketsemen, Marcello Iriti, Segun Gabriel Olanipekun

**Affiliations:** ^1^ Department of Chemistry Adamawa State University Mubi Adamawa Nigeria; ^2^ Department of Refining and Petrochemistry National Advanced School of Mines and Petroleum Industries University of Maroua Maroua Cameroon; ^3^ Department of Chemistry Adamawa State College of Education Hong Adamawa Nigeria; ^4^ Department of Organic Chemistry Faculty of Science University of Yaoundé I Yaoundé Cameroon; ^5^ Department of Biomedical Surgical and Dental Sciences University of Milan Milan Italy; ^6^ Department of Biochemistry Federal University of Technology Minna Nigeria

**Keywords:** antioxidant activity, essential oil, GC–MS, *Mercurialis perennis*, molecular docking, molecular dynamics simulation

## Abstract

*Mercurialis perennis* L. (Euphorbiaceae) is an underexplored perennial herb traditionally used in complementary medicine. This study provides a comprehensive investigation of its leaf essential oil, integrating chemical profiling, in vitro antioxidant evaluation, and computational analyses. The essential oil was obtained by steam distillation and analyzed using gas chromatography–mass spectrometry (GC–MS), leading to the identification of 31 compounds, predominantly sesquiterpene hydrocarbons, with *β*‐caryophyllene (58.14%) and γ‐muurolene (7.80%) as major constituents. The antioxidant activity was assessed using DPPH and hydrogen peroxide scavenging assays, yielding IC_50_ values of 7.15 ± 2.00 µg/mL and 0.42 ± 0.05 µg/mL, respectively. The results indicate moderate antioxidant activity of the essential oil. Molecular docking analysis suggested that γ‐muurolene may display favorable binding affinities toward selected oxidative stress‐related protein targets. Molecular dynamics simulations further indicated the stability of the 9h1m/γ‐muurolene complex, supported by persistent hydrophobic interactions with key residues (PHE41 and HIS170). Overall, these findings suggest that both major and minor constituents of the essential oil may contribute to its observed antioxidant activity. However, the computational results remain predictive and require further experimental validation. This study highlights the potential of *M. perennis* essential oil as a source of bioactive compounds and provides a basis for future in vivo and mechanistic investigations.

## Graphical abstract

Essential oil distilled from *Mercurialis perennis* leaves has been characterized by GC‐MS and assessed for their in vitro antioxidant activity.

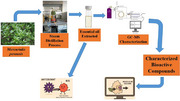

## 1 INTRODUCTION

*Mercurialis perennis* L. (Euphorbiaceae), commonly referred to as *Mercorella bastarda* [1], is a perennial herb that occurs broadly throughout temperate climatic zones. While the species is well documented in terms of its ecological role and phenotypic variability, its chemical composition remains insufficiently investigated. Members of the genus *Mercurialis* are known to exhibit marked interspecific differences in secondary metabolite production, indicating that *M. perennis* could constitute a largely untapped reservoir of biologically active constituents.

From a botanical perspective, *M. perennis* exhibits a dioecious reproductive system, with distinct male and female flowering structures [2, 3]. Although its morphological traits and reproductive mechanisms have been thoroughly documented, investigations focusing on its volatile profile and associated structure–activity relationships remain limited. Notably, the essential oil of *M. perennis* has yet to be comprehensively examined using contemporary analytical approaches.

Ethnomedicinal and locally documented uses of *M. perennis* provide further motivation for investigating its chemical profile. Traditional applications include topical treatment of wounds, burns, hemorrhoids, conjunctival irritation, and inflammatory conditions, as well as use in disorders related to blood pressure regulation, glucose metabolism, and skin pigmentation [4, 5, 6]. Historical records also indicate the use of *Mercurialis* species during the Middle Ages to address respiratory ailments, rheumatic conditions, and gynecological disorders. Despite this longstanding use, *M. perennis* is considered as potentially toxic when ingested, and detailed toxicological evaluations remain limited, highlighting the importance of molecular‐ and compound‐level investigations to better understand its biological effects [6, 7].

Oxidative imbalance is widely recognized as a key factor in the pathogenesis of numerous diseases. This condition is primarily associated with the activity of reactive oxygen and nitrogen species, which can initiate chain reactions leading to damage of lipids, proteins, carbohydrates, and nucleic acids [8, 9]. Antioxidants mitigate these effects by inhibiting or delaying oxidative processes, even at relatively low concentrations [8]. Due to the structural diversity of their constituents, particularly terpenoids and phenylpropanoids, essential oils have attracted attention as potential antioxidant systems acting through mechanisms such as hydrogen atom transfer, electron donation, and metal ion chelation.

However, it is important to note that commonly used in vitro antioxidant assays, including DPPH and hydrogen peroxide (H_2_O_2_) scavenging methods, present inherent limitations when applied to essential oils. The hydrophobic and volatile nature of essential oil components may result in solubility constraints and reduced interaction with assay reagents. In addition, nonspecific reactions and assay interference may lead to variability or misinterpretation of antioxidant capacity. Therefore, results obtained from these methods should be interpreted cautiously and considered as preliminary indicators of biological activity.

Building on this context, the present study investigates the essential oil obtained from *M. perennis* leaves through an integrated chemical and biological approach. The volatile composition was analyzed by GC–MS to determine qualitative and quantitative profiles. Antioxidant activity was evaluated using complementary in vitro assays targeting different mechanisms of redox activity. In parallel, molecular docking studies were conducted to explore potential interactions between selected constituents and protein targets associated with oxidative stress, providing insight into possible structure–activity relationships.

To the best of our knowledge, only limited studies have examined the essential oil of *M. perennis*, and data integrating chemical composition with both experimental antioxidant evaluation and computational analysis remain scarce. In this context, the present work aims to contribute to the existing body of knowledge by combining GC–MS characterization, in vitro antioxidant assays, molecular docking, and molecular dynamics simulations.

While molecular docking provides initial predictions of ligand–protein interactions, molecular dynamics simulations allow evaluation of the stability and dynamic behavior of these complexes over time. Accordingly, a 100 ns simulation was performed for the top‐ranked complex to assess binding stability, conformational flexibility, and the persistence of key intermolecular interactions under conditions approximating a physiological environment. Through this multidisciplinary approach, the study seeks to provide a more comprehensive understanding of the chemical profile and potential bioactivity of *M. perennis* essential oil.

## 2 Experimental

### 2.1 Sample Collection

Fresh leaf material of *M. perennis* L. was collected on 17^th^ March 2018 from bushland within the Sukur Settlement, Madagali Local Government Area, Adamawa State, Nigeria. The leaves were harvested from multiple trees within the same location, placed in labeled sacks, and stored in an ice cooler prior to transportation to the laboratory for extraction and analysis [10]. Botanical identification and taxonomic authentication were carried out by Prof. Dimas Kubmarawa (Natural Products Research Group), Modibbo Adama University, Yola.

### 2.2 Extraction of the Essential Oil

Fresh plant material (500 g per batch) was subjected to steam distillation using a Clevenger‐type apparatus following the method described in the British Pharmacopoeia (BP). The plant material was boiled in water, and volatile compounds were carried by steam, condensed, and collected in the Clevenger system, where the essential oil was separated from the aqueous phase based on immiscibility. Each distillation cycle was carried out for 2.5 h. The process was repeated until a total of 3.57042 kg of plant material had been distilled [11, 12].

The obtained essential oil was separated, dried over anhydrous sodium sulfate to remove residual moisture, and filtered. The oil yield was calculated and expressed as % (w/w) relative to the fresh weight of the plant material. The dried essential oil was stored in airtight amber glass vials at 4°C, protected from light, until further analysis.

### 2.3 Gas Chromatography Mass Spectrometry (GC/MS) Analysis of Essential Oil

The chemical composition of the essential oil was analyzed using an Agilent 190915‐433:469.56509 gas chromatograph coupled with a mass spectrometer (GC–MS). The system was equipped with a split/splitless injector operating at a pressure of 11.649 psi, with helium as the carrier gas at a constant flow rate of 1 mL/min. Separation was achieved on an HP‐5MS capillary column (30 m × 250 µm × 0.25 µm) coated with 5% phenylmethylsiloxane. The oven temperature was programmed from 60°C (held for 0.5 min) to 300°C at a rate of 10°C/min, followed by a final hold at 310°C for 22.5 min. The injector temperature was set at 310°C, and 0.2 µL of the sample was injected.

A homologous series of n‐alkanes (C_8–_C_20_) was analyzed under the same conditions to calculate retention indices (RI) for compound identification. Identification of constituents was based on comparison of mass spectra with library data and RI values from literature [10]. All analyses were performed in triplicate to ensure reproducibility. Quantification of the components was carried out using a semi‐quantitative approach based on normalized peak area percentages, without the application of correction factors.

### 2.4 Determination of Free Scavenging Activity of MPEO

#### 2.4.1 DPPH Scavenging Activity

The DPPH radical scavenging activity of the essential oil was evaluated according to the method of Burits and Bucar [13], with minor modifications. Briefly, 1 mL of the essential oil at concentrations ranging from 2.5 to 25 µg/mL (prepared in ethanol) was mixed with 4 mL of 0.004% DPPH solution in methanol. The mixture was incubated at room temperature for 30 min in the dark, and absorbance was measured at 517 nm using a spectrophotometer. Ascorbic acid (vitamin C) was used as the positive control.

The percentage inhibition of DPPH radicals was calculated using Equation (1):

(1) $$\mathrm{DPPH}\;\mathrm{Inhibition}\;\%=\frac{A_{\mathrm{Blank}}\;-\;A_{\mathrm{Sample}}}{A_{\mathrm{Blank}}}\;X\;100$$

Where A_Blank_ is the absorbance of the control reaction (without sample), and A_Sample_ is the absorbance in the presence of the essential oil.

#### 2.4.2 Hydrogen Peroxide Scavenging Activity

H_2_O_2_ scavenging activity was determined following the method described by Ruch et al. [14]. A 40 mM H_2_O_2_ solution was prepared in phosphate buffer (pH 7.4). An aliquot of 0.6 mL of this solution was mixed with varying concentrations of the essential oil (2.5–25 µg/mL) prepared in methanol. The absorbance was measured at 230 nm against a blank solution containing phosphate buffer without H_2_O_2_. Ascorbic acid was used as the reference standard. The percentage inhibition was calculated using Equation (2):

(2) $$\begin{array}{ccc} \mathrm{Hydrogen}\;\mathrm{peroxide}\;\mathrm{Inhibition}\;\% & = & \frac{A\;(\mathrm{control})\;-\;A\;(\mathrm{sample})}{A\;(\mathrm{control})}\\ & & \times 100 \end{array}$$

Where A_Control_ represents the absorbance of the control and A_Sample_ represents the absorbance in the presence of the essential oil.

### 2.5 Molecular Docking Analysis

#### 2.5.1 Preparation of Ligands

Three‐dimensional structures of selected compounds were constructed using Chem3D Pro 15.0 (PerkinElmer, USA). Energy minimization was performed using the MM2 force field to obtain stable conformations. The optimized structures were exported in Protein Data Bank (.pdb) format for docking studies.

#### 2.5.2 Preparation of Target Protein

Three protein targets associated with oxidative stress and antioxidant defense mechanisms were selected. The crystal structures of ascorbate peroxidase from soybean cytosol (PDB ID: 1oag) and recombinant ferric horseradish peroxidase C1A (PDB ID: 9h1m) were retrieved from the Protein Data Bank [15]. For the third target (PDB ID: 6xv4), a homology model was generated using the SWISS‐MODEL server [16], using the ascorbate peroxidase template (SMTL ID: 1oaf.1) based on sequence similarity. The generated model was validated using standard structural validation tools, including Ramachandran plot analysis, Verify3D, and ERRAT. All protein structures were prepared using AutoDockTools (ADT) version 1.5.6 [17]. Co‐crystallized ligands, water molecules, and ions were removed. Polar hydrogen atoms were added, and Gasteiger and Kollman charges were assigned. Protonation states were adjusted to physiological pH (7.4), followed by energy minimization.

#### 2.5.3 Molecular Docking Procedure

Docking simulations were carried out using AutoDock Vina version 1.2.7 [18]. Grid boxes were centered on the active sites of the proteins with the following coordinates: 1oag (x = 14.965, y = 54.235, z = 19.645), 6xv4 (x = 14.919, y = 55.758, z = 18.847), and 9h1m (x = 3.167, y = 30.855, z = 44.954). Each ligand was subjected to 50 independent docking runs, and binding poses were ranked according to binding energy (kcal/mol). The pose with the lowest binding energy was selected for further analysis. Validation of the docking protocol was performed by re‐docking the co‐crystallized ligand into the active site of each protein.

#### 2.5.4 Analysis of Molecular Interactions

Protein–ligand interactions were analyzed using Discovery Studio Visualizer 2021 (Dassault Systèmes BIOVIA, USA). Interactions such as hydrogen bonds, hydrophobic contacts, π–π interactions, and salt bridges were evaluated. Hydrogen bonds were defined by a donor–acceptor distance ≤ 3.5 Å and angle ≥ 120°. Binding affinities were expressed as binding energy values (kcal/mol).

### 2.6 Molecular Dynamics Simulation

#### 2.6.1 System Preparation and Equilibration

The docked 9h1m/γ‐muurolene complex was used as the starting structure. The system was solvated in a TIP3P water box with a 10 Å buffer and neutralized with Na^+^ and Cl^−^ ions. The ff14SB force field was applied to the protein, while GAFF2 parameters were assigned to the ligand. Energy minimization was performed in two stages: 5000 steps of steepest descent followed by 5000 steps of conjugate gradient minimization.

#### 2.6.2 Simulation Protocol

Molecular dynamics simulations were performed using PMEMD.CUDA in AmberTools22. The system was heated from 0 to 300 K over 100 ps under NVT conditions, followed by equilibration at 300 K and 1 atm for 1 ns under NPT conditions. A production run of 100 ns was conducted using a 2 fs time step. The Langevin thermostat and Monte Carlo barostat were applied. Trajectories were recorded every 50 ps.

#### 2.6.3 Trajectory Analysis

System stability was evaluated using root‐mean‐square deviation (RMSD) of protein Cα atoms and ligand. Ligand–protein interactions were analyzed by monitoring distances between the ligand and key residues (PHE41, HIS170, LEU37, MET281). Contact frequency was defined as the proportion of simulation time during which distances remained below 8.0 Å.

#### 2.6.4 Statistical Analysis

All in vitro experiments were conducted in triplicate, and results are expressed as mean ± standard deviation [19]. IC_50_ values were determined using nonlinear regression analysis (sigmoidal dose–response curve) with GraphPad Prism version 10.5.0.

## 3 Results and Discussion

### 3.1 MPEO Percentage Yield

A total of 3.57042 kg of fresh *M. perennis* leaves was processed via steam distillation to obtain the essential oil. The percentage yield of the oil, calculated according to Equation (3), is presented in Table 1.

(3) $$\%\;\mathrm{Yield}=\frac{\mathrm{Weight}\;\mathrm{of}\;\mathrm{oil}\;\left(\mathrm{WO}\right)}{\mathrm{Weight}\;\mathrm{of}\;\mathrm{plant}\;\left(\mathrm{WP}\right)}\times 100$$

**TABLE 1:** Percentage yield of essential oil.

Plant	Part used	WP (kg)	WO (g)	Appearance	% Yield
MP	Fresh leaves	3.57042	1.77	Pale green	0.05

Abbreviation: MP, M. perennis.

### 3.2 Antioxidant Activity

Oxidative reactions pose a major challenge to both fresh and processed foods, significantly limiting their shelf life and quality. Additionally, free radicals that cause biomolecular oxidation have been linked to the development of various diseases [20]. Amorati et al. [21] reported the efficiency of essential oil in neutralizing free radicals. Plant‐derived extracts have attracted attention as natural antioxidants due to their ability to neutralize reactive species and potentially contribute to food preservation and health benefits.

The antioxidant potential of *M. perennis* essential oil is summarized in Table 2. In the H_2_O_2_ scavenging assay, the oil exhibited an IC_50_ of 0.42 ± 0.05 µg/mL, whereas in the DPPH assay, the IC_50_ was 7.15 ± 2.00 µg/mL, indicating that activity varies depending on the type of radical system.

**TABLE 2:** Percentage Inhibition of MPEO and Ascorbic Acid against DPPH and H_2_O_2._

Assay	Conc. (µg/mL)/antioxidants	2.5	5	7.5	10
DPPH assay	MPEO (%)	43.10	47.18	65.29	81.81
	Vitamin C (%)	52.56	65.26	79.06	90.40
H_2_O_2_ assay	MPEO (%)	74.29	75.40	77.42	86.09
	Vitamin C (%)	79.13	86.59	88.71	90.12

**FIGURE 1:**
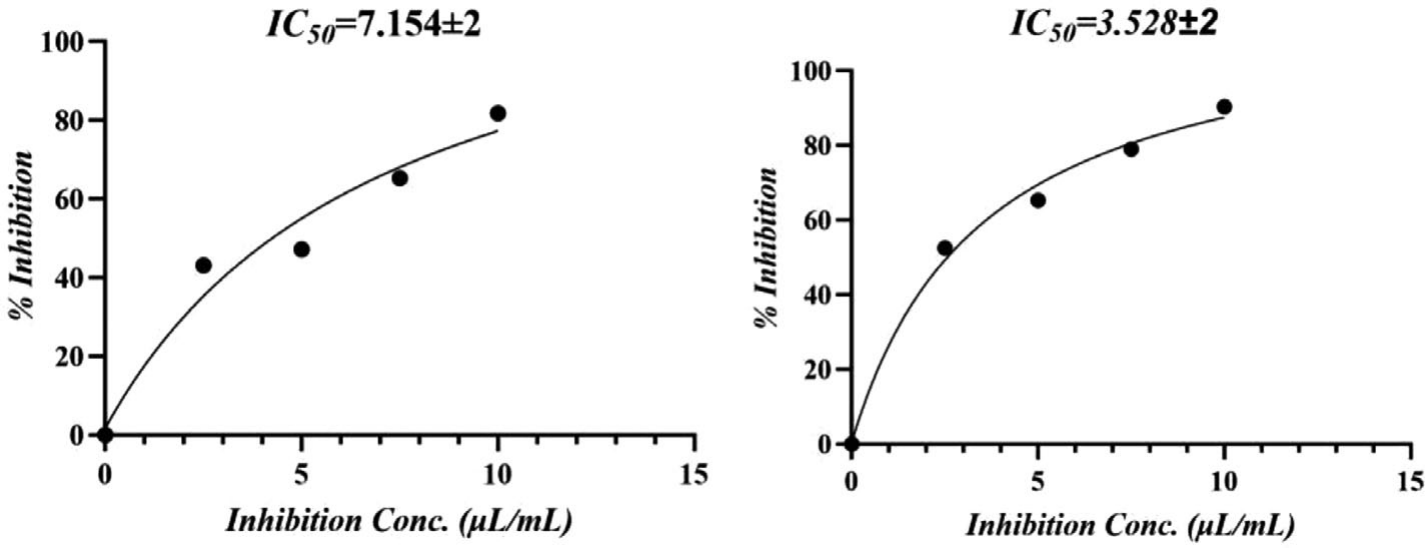
(a,b) IC_50_ curves of MPEO and ascorbic acid against DPPH radical.

**FIGURE 2:**
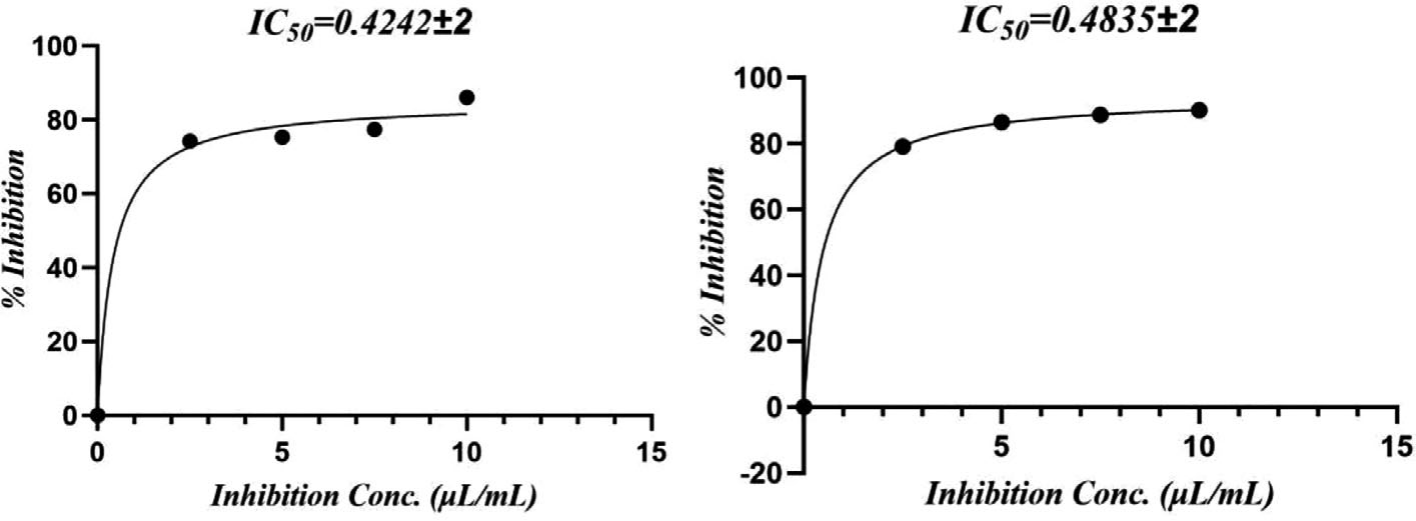
(a,b) IC_50_ curves of MPEO and ascorbic acid against H_2_O_2_ radical.

In the DPPH assay, ascorbic acid showed an IC_50_ of 3.53 ± 0.78 µg/mL compared with 7.15 ± 2.00 µg/mL for the essential oil (Figure 1). For H_2_O_2_ scavenging, the IC_50_ values were 0.484 ± 0.04 µg/mL for ascorbic acid and 0.422 ± 0.02 µg/mL for the oil (Figure 2). However, this apparent higher activity in the H_2_O_2_ assay should be interpreted cautiously, as differences in dose–response behavior and assay chemistry can influence IC_50_ estimation and do not necessarily imply superior antioxidant potency.

The antioxidant activity observed in essential oils is generally attributed to the presence of volatile terpenoids rather than phenolic compounds. In the present study, the oil is dominated by sesquiterpene hydrocarbons; therefore, attributing the activity to phenolics would not be appropriate. Instead, the activity is likely due to synergistic effects of terpenes and their ability to interact with reactive oxygen species through nonspecific mechanisms.

Although direct studies on *M. perennis* are scarce, related species in the Euphorbiaceae family have shown variable antioxidant activity depending on extract type and composition [22, 23, 24, 25, 26, 27, 28, 29, 30, 31]. These comparisons suggest that activity is strongly dependent on chemical composition, extraction method, and polarity of constituents.

### 3.3 GC–MS Analysis of the Essential Oil of *M. perennis* Leaves

**FIGURE 3:**
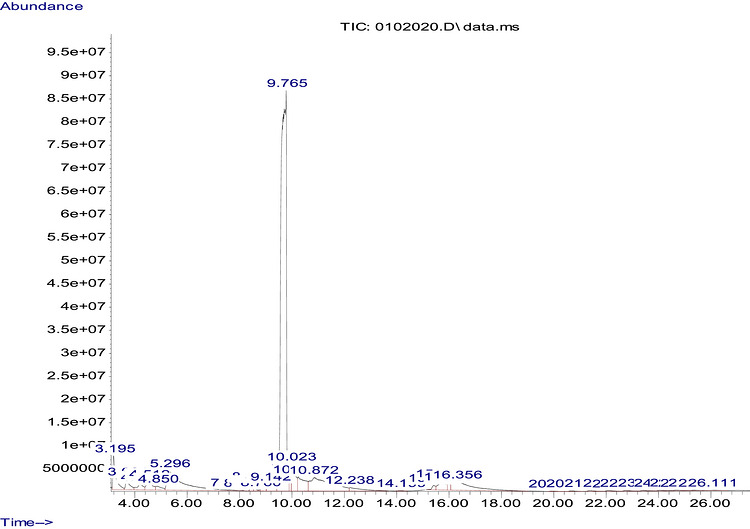
GC‐MS spectrum of essential oil extracted from *M. perennis*.

The GC–MS analysis of *M. perennis* leaf essential oil revealed 31 constituents, dominated by sesquiterpene hydrocarbons, particularly *β*‐caryophyllene (58.14%) (Figure 3 and Table 3). This confirms a sesquiterpene‐rich chemotype.

**TABLE 3:** GC‐MS essential oil analysis of leaves of *M. perennis*.

S/N	Constituents	Structural formula ^(^ ^a^ ^)^	Rt (Min)	Area (%)
1	*α*‐Pinene	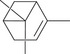	3.194	2.77
2	1,3,7‐Octatriene, 3,7‐dimethyl‐	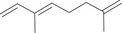	3.686	1.19
3	*α*‐Phellandrene	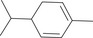	4.193	0.94
4	Limonene	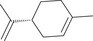	4.511	1.38
5	*γ*‐Terpinene	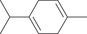	4.851	0.64
6	1‐Methyl‐4‐(6‐methylhept‐5‐en‐2‐yl)cyclohexa‐1,3‐diene	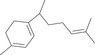	8.583	0.16
7	Copaene	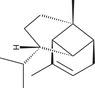	8.938	0.72
8	*β*‐Ocimene	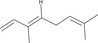	9.143	1.30
9	Caryophyllene	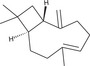	9.763	58.14
10	Isocaryophyllene	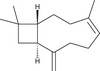	9.937	0.94
11	Humulene	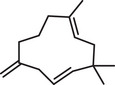	10.021	2.67
12	Santolina triene	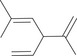	10.248	2.82
13	Cedrene	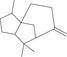	12.238	1.24
14	Cyclohexene, 1‐methyl‐4‐(1‐methyle thylidene)‐	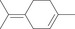	13.729	8.5
15	(*E*,*Z*)‐*α*‐Farnesene	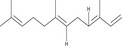	14.199	0.06
16	Alloaromadendrene	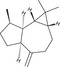	15.425	0.41
17	4‐(1‐prop‐1‐en‐2‐yl)cyclohex‐1‐en‐1‐yl)methanol	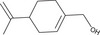	15.705	1.69
18	2‐Carene	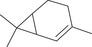	15.735	0.17
19	Camphene	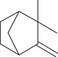	16.015	0.46
20	1,1,2‐Trimethyl‐3‐(2‐methylprop‐1‐en‐1‐ylidene)cyclopropane	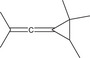	16.356	4.81
21	*γ*‐Muurolene	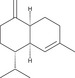	19.625	7.80
22	Octacosane	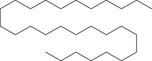	20.693	0.10
23	Hexatriacontane	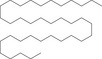	22.108	0.13
24	2‐methyltridecane	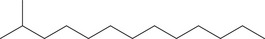	23.440	0.11
25	2‐methyleicosane	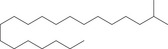	24.046	0.12
26	Vinyl lauryl ether	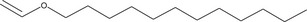	24.969	0.01
27	1‐Docosene	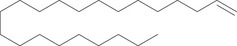	25.052	0.02
28	Hentriacontane	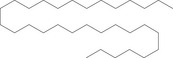	26.112	0.07
29	Tritetracontane	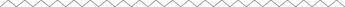	39.97	0.06
30	Eicosane	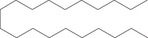	46.047	0.29
31	Octadecane	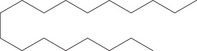	96.303	0.28
	**Total**			**100 %**

^a^ Chemical structures were drawn using ChemDraw Pro 8.0.

*β*‐caryophyllene and other sesquiterpenes such as γ‐muurolene and humulene have been previously associated with biological activities, including antioxidant and anti‐inflammatory effects [32]. However, it is important to note that essential oil bioactivity is typically multifactorial, resulting from combined and possibly synergistic effects of multiple constituents rather than a single dominant compound.

Unlike phenolic‐rich plant extracts, the present essential oil contains minimal oxygenated compounds. Therefore, antioxidant activity is unlikely to be driven by phenolic hydrogen donation mechanisms and is more consistent with terpene‐based radical quenching and membrane‐level interactions.

### 3.4 Molecular Docking Studies

**FIGURE 4:**
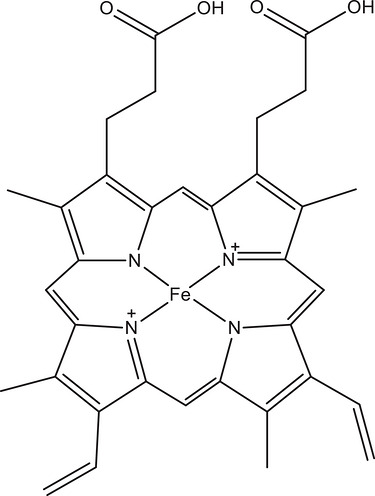
Protoporphyrin IX containing Fe, co‐crystalized ligand (L0) for 1oag, 6xv4 and 9h1m.

Molecular docking simulations demonstrated binding interactions between essential oil constituents and selected enzyme targets (Table 4). The co‐crystallized ligand (Protoporphyrin IX) (Figure 4) showed strong binding affinities across all protein targets, validating the docking protocol.

**TABLE 4:** Molecular docking scores and the corresponding prominent residual amino acid interactions of the major compounds against 1oag, 6xv4 and 9h1m enzymes.

Putative targets	Compounds	Binding energy (kcal/mol)	Key interacting residues and interaction types
1oag	Caryophyllene	−6.7	Alkyl—A chain: ALA134
	1‐methyl‐4‐(propan‐2‐ylidene)cyclohex‐1‐ene	−6.6	Alkyl and π‐Alkyl—A chain: TRP41, LEU159, HIS163, LEU242
	1,1,2‐trimethyl‐3‐(2‐methylprop‐1‐en‐1‐ylidene)cyclopropane	−5.3	π‐Sigma—A chain: TRP41
			Alkyl and π‐Alkyl—A chain: PHE145, LEU159
	ɤ‐Muurolene	−6.9	Alkyl and π‐Alkyl—A chain: LEU37, ARG38, TRP41, LEU159, HIS163, TYR235, HIS239, LEU242
	L0	−14.5	Attractive Charge—A chain: ARG38, ARG172
			Conventional Hydrogen Bond—A chain: ALA167, HIS169, SER173
			Carbon Hydrogen Bond—A chain: GLY166
			π‐Sigma—A chain: TRP41
			π‐π Stacked and π‐π Shaped‐ A chain: TRP179
			Alkyl and Pi‐Alkyl—A chain: LEU37, PRO132
6xv4	Caryophyllene	−6.6	Alkyl—A chain: ALA145
	1‐methyl‐4‐(propan‐2‐ylidene)cyclohex‐1‐ene	−6.5	Alkyl and π‐Alkyl—A chain: TRP52, LEU170, HIS174, LEU253
	1,1,2‐trimethyl‐3‐(2‐methylprop‐1‐en‐1‐ylidene)cyclopropane	−5.1	π‐Sigma—A chain: TRP52, HIS174
			Alkyl and π‐Alkyl—A chain: PHE156, PHE156, LEU170, HIS174
	ɤ‐Muurolene	−7.0	π‐Sigma—A chain: TRP52
			Alkyl and Pi‐Alkyl—A chain: PRO143, LEU170
	L0	−13.2	Attractive Charge—A chain: ARG49, ARG183
			Conventional Hydrogen Bond—A chain: ALA178, HIS180, SER184
			Carbon Hydrogen Bond—A chain: GLY177
			Metal‐Acceptor—A chain: HIS174
			π‐Sigma—A chain: TRP52
			π‐π Stacked and π‐π Shaped‐ A chain: TRP190
			Alkyl and π‐Alkyl—A chain: LEU48, PRO143, LEU170
9h1m	Caryophyllene	−6.6	Alkyl and π‐Alkyl—A chain: PRO141, LEU166, MET284
	1‐methyl‐4‐(propan‐2‐ylidene)cyclohex‐1‐ene	−7.1	Alkyl and π‐Alkyl—A chain: PHE41, PHE179
	1,1,2‐trimethyl‐3‐(2‐methylprop‐1‐en‐1‐ylidene)cyclopropane	−6.7	π‐Sigma—A chain: PHE41
			Alkyl and π‐Alkyl—A chain: LEU37, ARG38, LEU166, PHE277, MET281
	ɤ‐Muurolene	−8.4	Alkyl and Pi‐Alkyl—A chain: LEU37, ARG38, PHE41, LEU166, HIS170, PHE277, MET281, MET284
	L0	−15.4	Attractive Charge—A chain: ARG38
			Conventional Hydrogen Bond—A chain: SER35, SER73, LYS174, GLN176
			Metal‐Acceptor—A chain: HIS170
			π‐Cation—A chain: ARG31
			π‐Sigma—A chain: PHE41
			π‐π Stacked and π‐π Shaped‐ A chain: PHE221
			Alkyl and π‐Alkyl—A chain: LEU37, PRO139, LEU166

Among the tested compounds, *γ*‐muurolene showed the most favorable binding energies (–6.9 to –8.4 kcal/mol) (Table 5), followed by *β*‐caryophyllene. However, these binding values should be interpreted as computational predictions of binding tendency rather than direct evidence of biological inhibition.

**TABLE 5:** 3D and 2D binding interactions of ɤ‐Muurolene and L0 against 1oag, 6xv4 and 9h1m.

	Compounds			
Protein	ɤ‐Muurolene		L0	
	**2D**	**3D**	**2D**	**3D**
1oag	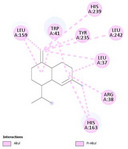	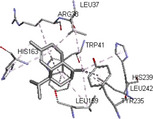	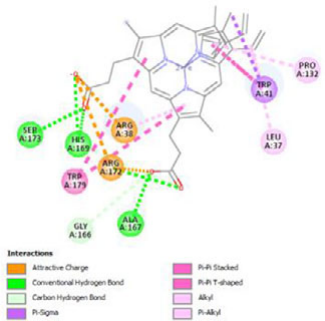	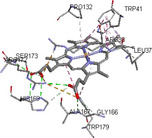
6xv4	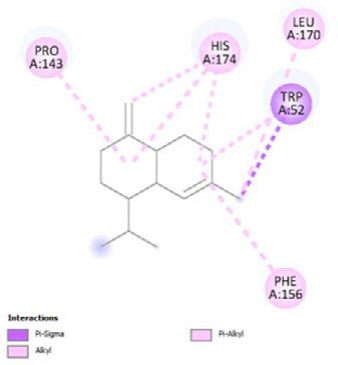	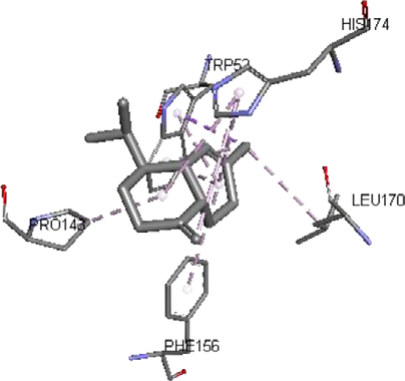	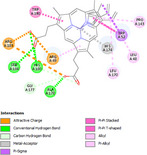	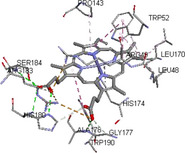
9h1m	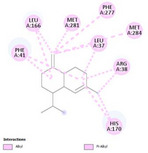	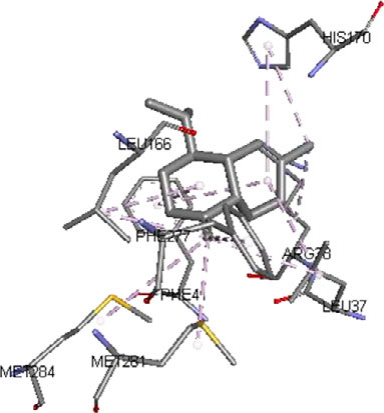	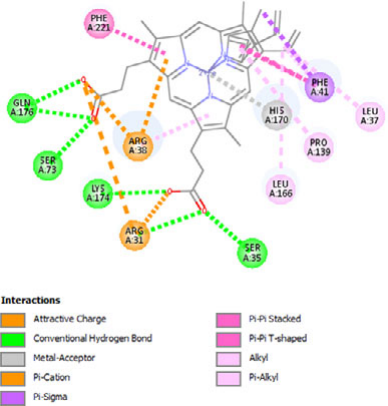	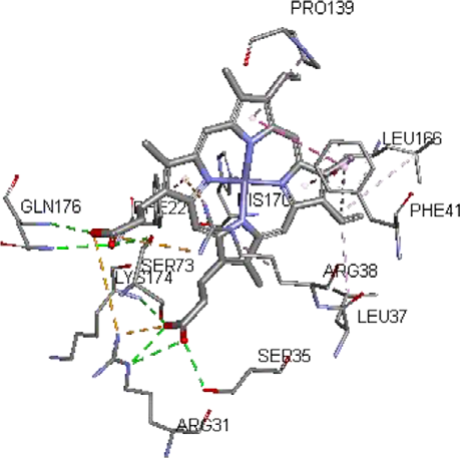

It is important to note that sesquiterpene hydrocarbons lack aromatic systems; therefore, interaction types reported as π–π stacking in docking outputs should not be interpreted literally. Instead, ligand binding is primarily driven by hydrophobic interactions, van der Waals forces, and steric complementarity within nonpolar binding pockets.

Although caryophyllene constituted the major component of the essential oil, the docking results indicated moderate binding affinities across all target proteins. The interactions were primarily driven by hydrophobic contacts (alkyl and π‐alkyl interactions in docking software terminology), van der Waals forces, and shape complementarity within the binding pocket.

### 3.5 Molecular Dynamics Validation of the 9h1M/γ‐Muurolene Complex

**FIGURE 5:**
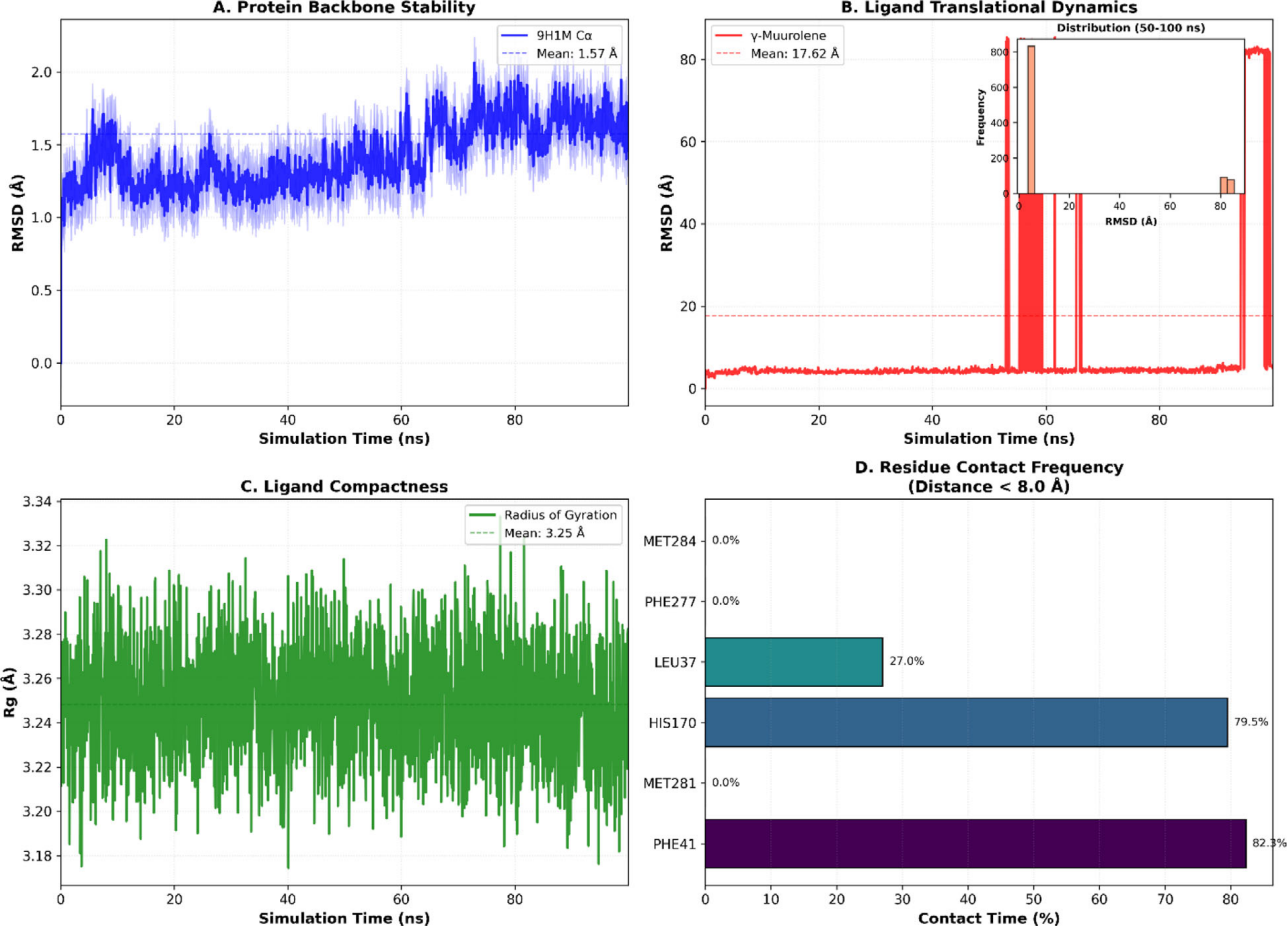
Structural dynamics of 9h1m/γ‐muurolene complex 100 ns molecular dynamics simulation.

To validate the docking‐predicted binding mode of γ‐muurolene within the 9h1m active site, a 100 ns MD simulation was performed. The protein backbone remained stable throughout the simulation, with an average Cα RMSD of 1.57±0.17 Å during the last 50 ns (Figure 5A), indicating structural integrity of the receptor under physiological conditions. In contrast, γ‐muurolene exhibited relatively high RMSD fluctuations (17.62 ± 29.39 Å) (Figure 5B), reflecting substantial conformational mobility within the solvent environment and partial retention–dissociation behavior within the binding region. This behavior suggests that the ligand does not form a rigid or permanently stable complex within the binding pocket. Instead, the interaction is better described as transient and reversible hydrophobic association, which is typical for small nonpolar terpenoid molecules that lack strong directional interactions.

**FIGURE 6:**
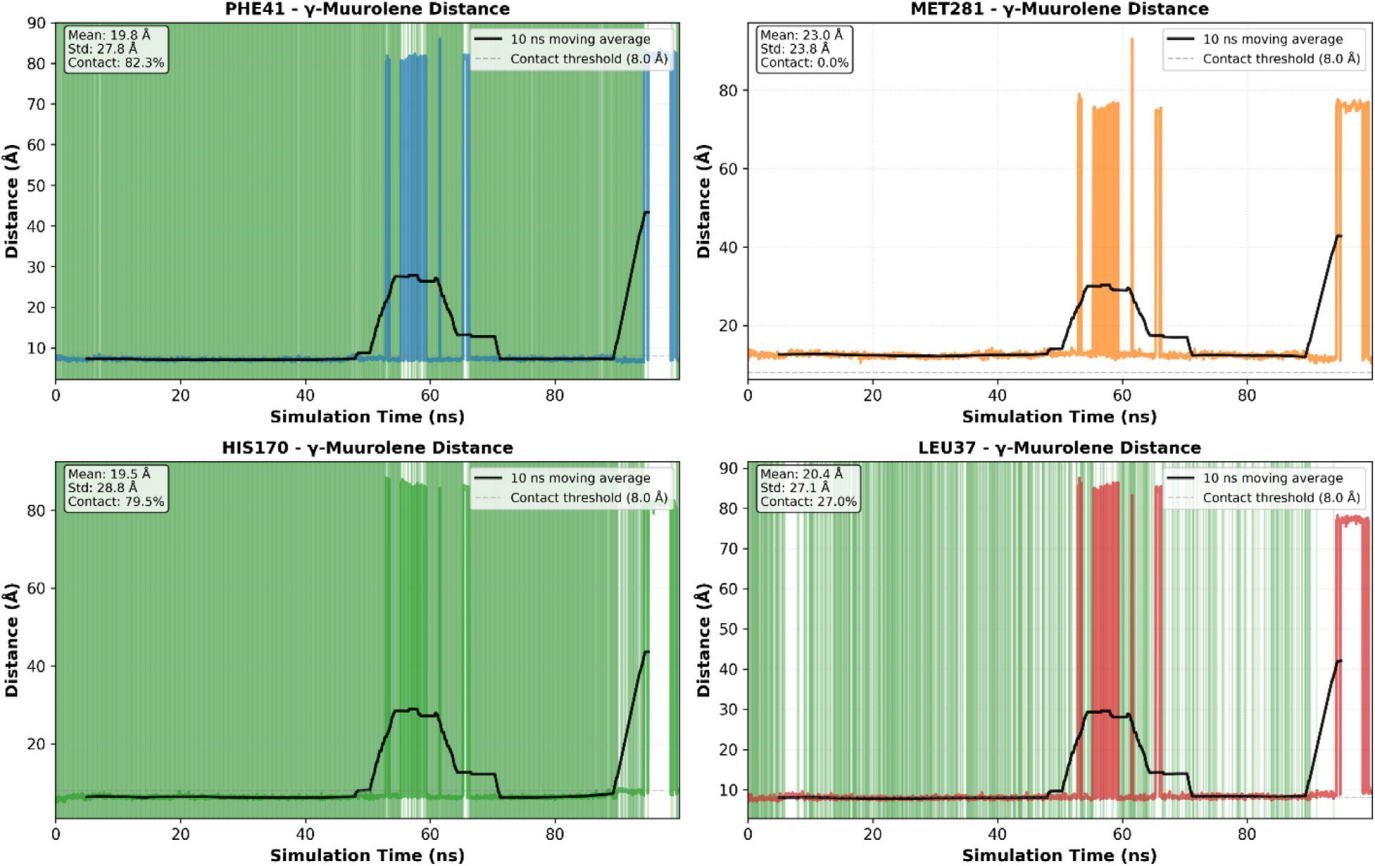
Evolution of ligand‐residue distances, hydrophobic interaction dynamics.

**FIGURE 7:**
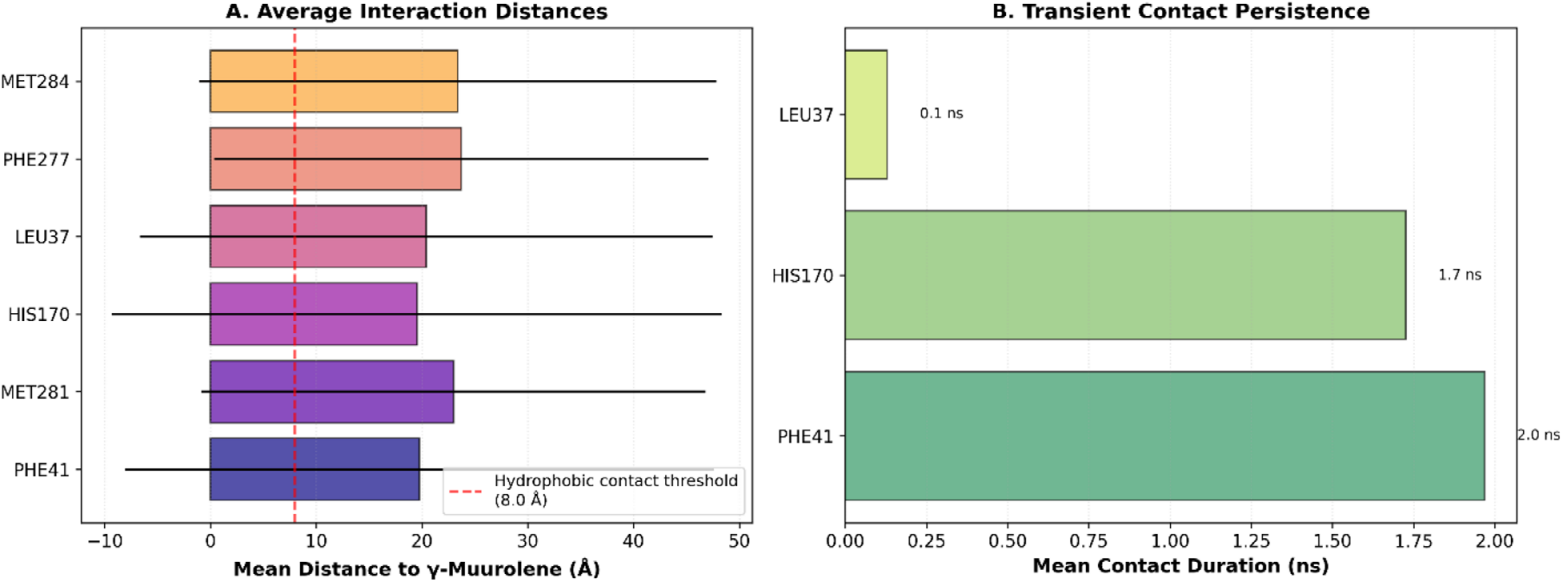
Hydrophobic ligand behavior in MD simulation.

Despite this mobility, the ligand showed intermittent proximity to key residues such as PHE41 and HIS170 (Figures 5D, 6, and 7), suggesting that these residues contribute to weak and temporary recognition events. However, these contacts were not sufficiently stable to indicate persistent binding.

Therefore, molecular dynamics results refine the docking predictions by demonstrating that although a hydrophobic pocket exists, ligand binding is dynamic and short‐lived rather than stable.

## 4 Conclusion

The present study investigated the chemical composition and antioxidant potential of *M. perennis* leaf essential oil. GC–MS analysis revealed a sesquiterpene‐rich profile dominated by *β‐*caryophyllene, indicating a characteristic volatile composition for this species. The essential oil demonstrated measurable antioxidant activity in both DPPH and H_2_O_2_ scavenging assays, with variation depending on the radical system tested. These results indicate that the oil contains constituents capable of interacting with different reactive oxygen species under in vitro conditions. Molecular docking analysis suggested that certain constituents, particularly γ‐muurolene, may interact with oxidative stress‐related protein targets mainly through hydrophobic interactions. However, these findings should be considered predictive computational indications rather than evidence of confirmed biological mechanisms. Molecular dynamics simulation further indicated that the interaction between γ‐muurolene and the 9h1m protein is dynamic and transient, characterized by reversible contacts with key residues such as PHE41 and HIS170 rather than stable binding. Overall, the results suggest that the observed antioxidant activity of *M. perennis* essential oil is likely associated with the combined effects of its sesquiterpene constituents. Nevertheless, further experimental studies, including bioassay‐guided fractionation and in vivo validation, are required to better understand the biological relevance and mechanisms of action.

## Supplementary Information

Additional files‐Only the best‐scoring docking pose for each protein is presented in this article, the complete set of docking results, including all 2D and 3D structures, is provided as Supporting Information.

## Author Contributions

**Chika Attama**: conceptualization, resources, methodology, investigation, formal analysis, data curation, writing – original draft, validation. **Jean Noël Nyemb**: conceptualization, methodology, formal analysis, data curation, writing – original draft, writing – review and editing, validation, supervision. **Lawrence Luka**: methodology, investigation, data curation, writing – review and editing. **Herve Landry Ketsemen**: methodology, investigation, formal analysis, software, writing – original draft. **Marcello Iriti**: resources, visualization, validation, funding acquisition, supervision. **Segun Gabriel Olanipekun**: conceptualization, resources, validation, supervision and writing – review and editing.

## Funding

The authors have nothing to report.

## Conflicts of Interest

The authors declare no conflicts of interest.

## Supporting information




**Supporting File 1**: cbdv71337‐sup‐0001‐SuppMat.pdf

**Supporting File 2**: cbdv71337‐sup‐0002‐SuppMat.pdf

## Data Availability

Data supporting the conclusion of this research are presented in the tables and figures in this article.

## References

[1] L. De Simone , S. Santoro , and C. Guarino , “Ethnobotanical Study of the Sannio Area, Campania, Southern Italy,” Ethnobotany Research and Applications 6 (2008): 255, 10.17348/era.6.0.255-317.

[2] V. Vujić , L. Rubinjoni , S. Selaković , and D. Cvetković , “Small‐Scale Variations in Leaf Shape Under Anthropogenic Disturbance in Dioecious Forest Forb *Mercurialis perennis*: A Geometric Morphometric Examination,” Archives of Biological Sciences 68 (2016): 705–713, 10.2298/ABS151111011V.

[3] J. Blanco‐Salas , F. M. Vazquez , M. P. Hortigón‐Vinagre , and T. Ruiz‐Tellez , “Bioactive Phytochemicals From *Mercurialis* spp. Used in Traditional Spanish Medicine,” Plants 8, no. 7 (2019): 193, 10.3390/plants8070193. 31261793 PMC6681364

[4] P. Lorenz , M. Bunse , S. Sauer , J. Conrad , F. C. Stintzing , and D. R. Kammerer , “Conversion of Plant Secondary Metabolites Upon Fermentation of *Mercurialis perennis* L. Extracts With Two Lactobacteria Strains,” Fermentation 5 (2019): 42, 10.3390/fermentation5020042.

[5] P. Lorenz , M. Knödler , J. Bertrams , M. Berger , U. Meyer , and F. C. Stintzing , “n‐Alkylresorcinol Occurrence in *Mercurialis perennis* L. (Euphorbiaceae),” Zeitschrift für Naturforschung C 65 (2010): 174–179, 10.1515/znc-2010-3-402. 20469634

[6] N. Mut‐Salud , P. J. Álvarez , J. M. Garrido , E. Carrasco , A. Aránega , and F. Rodríguez‐Serrano , “Antioxidant Intake and Antitumor Therapy: Toward Nutritional Recommendations for Optimal Results,” Oxidative Medicine and Cellular Longevity 6719534 (2015): 1–20, 10.1155/2016/6719534. PMC4670692 26682013

[7] T. I. Mbata , “Antioxidant Nutrients: Beneficial or Harmful,” Internet Journal of Food Safety 7 (2005): 29–33.

[8] P. Lorenz , I. Zilkowski , L. K. Mailänder , et al., “Comparison of Aqueous and Lactobacterial‐Fermented *Mercurialis perennis* L. (Dog's Mercury) Extracts With Respect to Their Immunostimulating Activity,” Fermentation 9 (2023): 190, 10.3390/fermentation9020190.

[9] D. Kubmarawa , G. A. Ajoku , N. M. Enwerem , and D. A. Okorie , “Preliminary Phytochemical and Antimicrobial Screening of 50 Medicinal Plants From Nigeria,” African Journal Biotechnology 6, no. 14 (2007): 1690–1696.

[10] Z. Iqbal , A. Saeed , M. Mansha , et al., “Essential Oil From the Aerial Roots of *Ficus elastica* and Their Antioxidant Activity,” International Journal of Advanced Research 6, no. 1 (2018): 137–140, 10.21474/IJAR01/6195.

[11] M. Burits and F. Bucar , “Antioxidant Activity of *Nigella sativa* Essential Oil,” Phytotherapy Research 14, no. 15 (2000): 323–328, 10.1002/1099-1573(200008)14:5<323::AID-PTR621>3.0.CO;2-Q. 10925395

[12] N. Al‐Douri , B. A. Al‐Jaidi , H. M. Hammad , et al., “Biological Evaluation of *Mercurialis annua* Extracts for Possible Antioxidant, Antiproliferative and Cytotoxic Activity,” Indian Journal of Pharmaceutical Education and Research 56, no. 3s (2022): s479–s486, 10.5530/ijper.56.3s.156.

[13] N. Al‐Douri and A. K. Shakya , “Fatty Acids Analysis and Antioxidant Activity of a Lipid Extract Obtained From *Mercurialis annua* L. Grown Wildly in Jordan,” Natural Product Research 72, no. 2 (2019): 275–281, 10.32383/appdr/97344.

[14] O. A. Rugaie , H. A. Mohammed , S. Alsamani , et al., “Antimicrobial, Antibiofilm, and Antioxidant Potentials of Four Halophytic Plants, *Euphorbia chamaesyce*, *Bassia arabica*, *Fagonia mollis*, and *Haloxylon Salicornicum*, Growing in Qassim Region of Saudi Arabia: Phytochemical Profile and In Vitro and In Silico Bioactivity Investigations,” Antibiotics 12 (2023): 501, 10.3390/antibiotics12030501. 36978368 PMC10044527

[15] E. Simionatto , V. F. L. Bonani , A. F. Morel , et al., “Chemical Composition and Evaluation of Antibacterial and Antioxidant Activities of the Essential Oil of *Croton urucurana* Baillon (Euphorbiaceae) Stem Bark,” Journal of the Brazilian Chemical Society 18, no. 5 (2007): 879–885, 10.1590/S0103-50532007000500002.

[16] D. U. Meenakshi , T. Alam , R. Zafarullah , and S. Khan , “Chemical Composition and In Vitro Biological Evaluation of Essential Oil of *Euphorbia larica* Boiss From Northern Oman,” Asian Journal of Chemistry 35, no. 8 (2023): 1879–1883, 10.14233/ajchem.2023.27948.

[17] A. Rahman , M. Rahman , and I. Demirtas , “Chemical Composition and Antioxidant Potential of Essential Oil and Organic Extracts of *Euphorbia tithymaloides* L. From Kushtia Region,” Anti‐Cancer Agents in Medicinal Chemistry 18, no. 10 (2018): 1482–1488, 10.2174/1871520618666180228122318. 29493467

[18] A. Kadri , N. Gharsallah , N. Damak , and R. Gdoura , “Chemical Composition and In Vitro Antioxidant Properties of Essential Oil of *Ricinus communis* L,” Journal of Medicinal Plants Research 5 (2011): 1466–1470, 10.5897/JMPR.9000347.

[19] P. A. Onocha , G. K. Oloyede , and Q. O. Afolabi , “Chemical Composition, Cytotoxicity and Antioxidant Activity of Essential Oils of *Acalypha hispida* Flowers,” International Journal of Pharmacology 7 (2011): 144–148, 10.3923/ijp.2011.144.148.

[20] M. V. Cetiz , S. Ahmed , G. Zengin , et al., “Bioinformatic and Experimental Approaches to Uncover the Bio‐Potential of *Mercurialis annua* Extracts Based on Chemical Constituents,” Journal of Molecular Liquids 427 (2025): 127390, 10.1016/j.molliq.2025.127390.

[21] A. Siddiq , F. Anwar , M. Manzoor , and A. Fatima , “Antioxidant Activity of Different Solvent Extracts of *Moringa oleifera* Leaves Under Accelerated Storage of Sunflower Oil,” Asian Journal of Plant Sciences 4 (2005): 630–635, 10.3923/ajps.2005.630.635.

[22] Y. Masui , H. Kameoka , K. Amano , and K. Abe , “Constituents of the Essential Oil of *Mercurialis leiocarpa* ,” Nippon Nogeikagaku Kaishi 62, no. 8 (1988): 1217–1219.

[23] M. S. Owolabi , A. O. Ogundajo , I. A. Ogunwande , R. M. Hauser , and W. N. Setzer , “Essential Oil Composition of *Caperonia palustris* (L.) A. St‐Hil. (Euphorbiaceae) Growing in South West, Nigeria,” American Journal of Essential Oils and Natural Products 2, no. 1 (2014): 39–40.

[24] C. N. Ginting , I. N. Lister , E. Girsang , et al., “In Silico Anti‐Preeclampsia Potential of Phytochemical Found in *Ficus elastica* ,” Pharmacognosy Research 11 (2019): 279, 10.4103/pr.pr_176_18.

[25] L. Nogueira de M , M. R. da Silva , S. M. Dos Santos , et al., “Antinociceptive Effect of the Essential Oil Obtained From the Leaves of *Croton cordiifolius* Baill. (Euphorbiaceae) in Mice,” Evidence‐Based Complementary and Alternative Medicine 2015 (2015): 620865, 10.1155/2015/620865. 25821494 PMC4363708

[26] Y. Yukio , K. Nakamura , and M. Sato , “Chemical Composition of the Essential Oil of *Mercurialis leiocarpa* Sieb. Et Zucc,” Journal of Essential Oil Research 17 (2005): 45–50.

[27] K. H. C. Baser and G. Buchbauer , Handbook of Essential Oils: Science, Technology, and Applications (CRC Press, 2010).

[28] C. Giuliani , L. M. Bini , M. M. Lippi , and M. Innocenti , “Essential Oil Composition of Several *Croton* Species From the Euphorbiaceae Family,” Flavour and Fragrance Journal 27 (2012): 321–327.

[29] M. A. Lenise , E. V. Costa , and P. L. B. Figueiredo , “Chemical Composition of the Essential Oil of *Croton cordiifolius* ,” Natural Product Communications 9 (2014): 1045–1048.25230523

[30] R. Sita , O. Prakash , and A. K. Pant , “GC–MS Analysis of the Essential Oil of Croton pallidulus,” Journal of Essential Oil Bearing Plants 19 (2016): 1123–1129.

[31] E. O. Moses , A. J. Afolayan , and M. O. Sofidiya , “Chemical Composition of the Essential Oil From the Aerial Parts of *Caperonia palustris* ,” Asian Journal of Chemistry 23 (2011): 2569–2572.

[32] J. Gertsch , M. Leonti , S. Raduner , et al., “Beta‐Caryophyllene is a Dietary Cannabinoid,” Proceedings of the National Academy of Sciences of the United States of America 105 (2008): 9099–9104, 10.1073/pnas.0803601105. 18574142 PMC2449371

