# Supplementary material for: β‐Caryophyllene‐Rich Mercurialis perennis Leaf Essential Oil: GC–MS Profiling, Antioxidant Activity, Molecular Docking, and Molecular Dynamics Analysis
Source: Chem Biodivers. 2026 May 15;23:e71337. doi: 10.1002/cbdv.71337 (PMC13178399; doi:10.1002/cbdv.71337)
Supplement: Supplementary file 1 — Supporting File 1: cbdv71337‐sup‐0001‐SuppMat.pdf [file CBDV-23-e71337-s001.pdf]

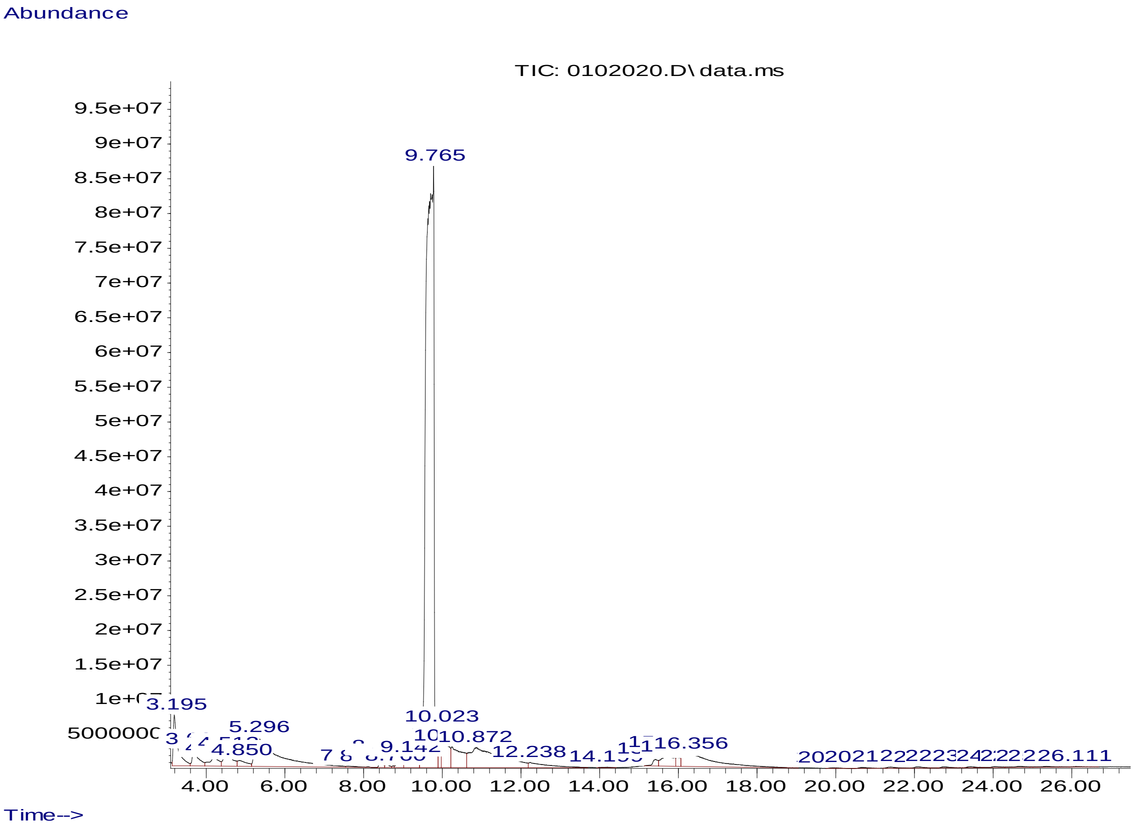

Chemistry Lab

Library Search Report

Data Path : C:\Users\chemistrylab\Desktop\1\DATA\0101677.D\

Data File : 0102020.D

Acq On : 25 Sep 2019 17:35

Operator :

Sample : L

Misc :

ALS Vial : 1 Sample Multiplier: 1

Search Libraries: C:\Database\NIST11.L Minimum Quality: 50

C:\Database\NIST14.L Minimum Quality: 0

Unknown Spectrum: Apex

Integration Events: ChemStation Integrator - autoint1.e

Pk#	RT	Area%	Library/ID	Ref#	CAS#	Qual

1	3.194	2.77	C:\Database\NIST11.L			
			(1R)-2,6,6-Trimethylbicyclo[3.1.1]hept-2-ene	15854	007785-70-8	96
			.alpha.-Pinene	15699	000080-56-8	95
			(1R)-2,6,6-Trimethylbicyclo[3.1.1]hept-2-ene	15852	007785-70-8	94
2	3.686	1.19	C:\Database\NIST11.L			
			1,3,7-Octatriene, 3,7-dimethyl-	15769	000502-99-8	91
			(1R)-2,6,6-Trimethylbicyclo[3.1.1]hept-2-ene	15854	007785-70-8	91
			Cyclohexene, 4-methylene-1-(1-methylethyl)-	15844	000099-84-3	90
3	4.193	0.94	C:\Database\NIST11.L			
			.alpha.-Phellandrene	15728	000099-83-2	91
			.alpha.-Phellandrene	15730	000099-83-2	91
			Bicyclo[3.1.0]hex-2-ene, 2-methyl-5-(1-methylethyl)-	15898	002867-05-2	90
4	4.511	1.38	C:\Database\NIST11.L			
			4-Carene, (1S,3R,6R)-(-)-	15741	005208-49-1	64
			D-Limonene	15682	005989-27-5	64
			Bicyclo[3.1.1]hept-2-ene, 3,6,6-trimethyl-	15837	004889-83-2	64
5	4.851	0.64	C:\Database\NIST11.L			
			.gamma.-Terpinene	15711	000099-85-4	94
			3-Carene	15664	013466-78-9	91
			(1S)-2,6,6-Trimethylbicyclo[3.1.1]hept-2-ene	15851	007785-26-4	87

```
 6   5.298  8.13 C:\Database\NIST11.L
                Cyclohexene, 1-methyl-4-(1-methyle  15862 000586-62-9 95
                thylidene)-
                Cyclohexene, 1-methyl-4-(1-methyle  15863 000586-62-9 95
                thylidene)-
                Cyclohexene, 1-methyl-4-(1-methyle  15858 000586-62-9 94
                thylidene)-

 7   7.614  0.14 C:\Database\NIST11.L
                Cyclohexene, 1-methyl-4-(1-methyle  15858 000586-62-9 96
                thylidene)-
                2-Carene                            15666 000554-61-0 87
                (+)-4-Carene                        15688 029050-33-7 87

 8   8.121  0.03 C:\Database\NIST11.L
                Cyclohexene, 1-methyl-4-(1-methyle  15858 000586-62-9 95
                thylidene)-
                2-Carene                            15666 000554-61-0 76
                1,3-Cyclohexadiene, 1-methyl-4-(1-  15877 000099-86-5 76
                methylethyl)-

 9   8.431  0.37 C:\Database\NIST11.L
                Cyclohexene, 4-ethenyl-4-methyl-3-  64501 020307-84-0 98
                (1-methylethenyl)-1-(1-methylethyl
                )-, (3R-trans)-
                2-Carene                            15666 000554-61-0 91
                Cyclohexene, 1-methyl-4-(1-methyle  15863 000586-62-9 90
                thylidene)-

10   8.583  0.16 C:\Database\NIST14.L
                1-Methyl-4-(6-methylhept-5-en-2-yl  68678 000451-55-8 89
                )cyclohexa-1,3-diene
                Cyclohexene, 1-methyl-4-(1-methyle  16238 000586-62-9 64
```

```
               thylidene)-
               Spiro[5.5]undeca-1,8-diene, 1,5,5,  68681 019912-83-5 52
               9-tetramethyl-, (R)-

11   8.757  0.04 C:\Database\NIST11.L
               .gamma.-Muurolene                   64338 030021-74-0 62
               .gamma.-Muurolene                   64337 030021-74-0 58
               Cyclohexene, 1-methyl-4-(1-methyle  15858 000586-62-9 52
               thylidene)-

12   8.938  0.72 C:\Database\NIST11.L
               Copaene                             64250 003856-25-5 94
               Copaene                             64252 003856-25-5 93
               .alpha.-ylangene                    64307 1000374-19-0 64

13   9.143  1.30 C:\Database\NIST11.L
               1,3,6-Octatriene, 3,7-dimethyl-, (  15808 003338-55-4 50
               Z)-
               .beta.-Ocimene                      15698 013877-91-3 49
               1H-Pyrrole, 1-butyl-                10219 000589-33-3 46

14   9.763 58.14 C:\Database\NIST11.L
               Caryophyllene                       64272 000087-44-5 99
               Caryophyllene                       64275 000087-44-5 97
               Bicyclo[5.2.0]nonane, 2-methylene-  64421 242794-76-9 95
               4,8,8-trimethyl-4-vinyl-

15   9.937  0.94 C:\Database\NIST11.L
               Isocaryophillene                    64304 1000140-07-2 86
               Bicyclo[7.2.0]undec-4-ene, 4,11,11  64479 000118-65-0 83
               -trimethyl-8-methylene-,[1R-(1R*,4
               Z,9S*)]-
               .alpha.-ylangene                    64307 1000374-19-0 78
```

16 10.021 2.67 C:\Database\NIST11.L

Bicyclo[3.1.1]hept-2-ene, 2,6-dime 64434 017699-05-7 83

thyl-6-(4-methyl-3-pentenyl)-

cis-.alpha.-Bisabolene 64361 029837-07-8 72

Humulene 64256 006753-98-6 70

17 10.248 2.82 C:\Database\NIST11.L

Santolina triene 15707 002153-66-4 83

Bicyclo[7.2.0]undec-4-ene, 4,11,11 64477 000118-65-0 62

-trimethyl-8-methylene-,[1R-(1R*,4

Z,9S*)]-

1,7-Octadiene, 2,7-dimethyl-3,6-bi 32335 016714-60-6 62

s(methylene)-

18 10.868 7.76 C:\Database\NIST11.L

Naphthalene, 1,2,4a,5,6,8a-hexahyd 64456 000483-75-0 97

ro-4,7-dimethyl-1-(1-methylethyl)-

.gamma.-Muurolene 64337 030021-74-0 96

.gamma.-Muurolene 64324 030021-74-0 91

19 12.238 1.24 C:\Database\NIST11.L

Cedrene 64249 011028-42-5 83

Tricyclo[5.4.0.0(2,8)]undec-9-ene, 64464 005989-08-2 81

2,6,6,9-tetramethyl-, (1R,2S,7R,8

R)-

1H-Benzocycloheptene, 2,4a,5,6,7,8 64507 003853-83-6 78

,9,9a-octahydro-3,5,5-trimethyl-9-

methylene-, (4aS-cis)-

20 14.199 0.06 C:\Database\NIST11.L

(E,Z)-.alpha.-Farnesene 64366 1000293-03-2 58

cis-.alpha.-Bisabolene 64361 029837-07-8 58

2,5-Cyclooctadiene-1-carboxamide, 83637 1000159-75-2 50
N-phenyl-

21 15.425 0.41 C:\Database\NIST11.L
1,3,6,10-Cyclotetradecatetraene, 3 121356 001898-13-1 96
,7,11-trimethyl-14-(1-methylethyl)
-, [S-(E,Z,E,E)]-
Alloaromadendrene 64332 025246-27-9 70
1,5-Cycloundecadiene, 8,8-dimethyl 53193 062338-54-9 64
-9-methylene-

22 15.705 1.69 C:\Database\NIST11.L
1-Cyclohexene-1-methanol, 4-(1-met 25281 000536-59-4 50
hylethenyl)-
1-Nitro-bicyclo[6.1.0]nonan-2-one 48257 1000282-07-3 46
2,3-Hexadiene, 2-methyl- 2886 029212-09-7 43

23 16.015 0.46 C:\Database\NIST11.L
1,3,6,10-Cyclotetradecatetraene, 3 121356 001898-13-1 91
,7,11-trimethyl-14-(1-methylethyl)
-, [S-(E,Z,E,E)]-
Cyclohexane, 1-ethenyl-1-methyl-2, 64415 110823-68-2 78
4-bis(1-methylethenyl)-
Camphene 15676 000079-92-5 38

24 16.356 4.81 C:\Database\NIST11.L
Cyclopropane, trimethyl(2-methyl-1 15889 014803-30-6 64
-propenylidene)-
1,3,6,10-Cyclotetradecatetraene, 3 121356 001898-13-1 56
,7,11-trimethyl-14-(1-methylethyl)
-, [S-(E,Z,E,E)]-
Bicyclo[4.1.0]heptane, 7-(1-methyl 15866 053282-47-6 46
ethylidene)-

25	19.951	0.03	C:\Database\NIST11.L			
			Dodecane, 1,2-dibromo-	165284	055334-42-4	64
			Decane, 1-iodo-	116706	002050-77-3	55
			Tritetracontane	241174	007098-21-7	52
26	20.019	0.03	C:\Database\NIST14.L			
			Octadecane, 1-chloro-	148106	003386-33-2	49
			1-Octadecanesulphonyl chloride	206960	1000342-70-4	49
			Tritetracontane	273205	007098-21-7	46
27	20.693	0.10	C:\Database\NIST11.L			
			Nonadecane	117636	000629-92-5	90
			Octacosane	209566	000630-02-4	83
			Methoxyacetic acid, 2-tetradecyl e ster	132964	1000282-04-8	83
28	21.396	0.13	C:\Database\NIST11.L			
			Eicosane	129493	000112-95-8	89
			Heneicosane, 11-pentyl-	194505	014739-72-1	87
			Tricosane, 2-methyl-	175565	001928-30-9	83
29	22.108	0.13	C:\Database\NIST11.L			
			Hexatriacontane	235974	000630-06-8	87
			Tricosane, 2-methyl-	175564	001928-30-9	87
			Docosane, 9-octyl-	219832	055319-83-0	87
30	22.456	0.01	C:\Database\NIST11.L			
			Octadecane, 1-chloro-	134595	003386-33-2	55
			Octadecane	105884	000593-45-3	55
			Nonadecane	117638	000629-92-5	55
31	22.751	0.12	C:\Database\NIST11.L			

```
                Octadecane                       105885 000593-45-3 97
                Octadecane                       105884 000593-45-3 96
                Octadecane                       105883 000593-45-3 95

32  23.440  0.11 C:\Database\NIST11.L
                Tridecane, 2-methyl-              59889 001560-96-9 87
                1-Iodo-2-methylundecane          140553 073105-67-6 78
                Octadecane, 2-methyl-            117649 001560-88-9 70

33  24.046  0.12 C:\Database\NIST11.L
                Eicosane, 2-methyl-              141430 001560-84-5 90
                Octadecane                       105883 000593-45-3 90
                1-Chloroeicosane                 158254 042217-02-7 90

34  24.651  0.16 C:\Database\NIST11.L
                Eicosane                         129492 000112-95-8 87
                Hexadecane, 1-chloro-            110906 004860-03-1 86
                Tricosane, 2-methyl-             175566 001928-30-9 70

35  24.969  0.01 C:\Database\NIST11.L
                .alpha.-Ketostearic acid         143037 004468-17-1 83
                2-Piperidinone, N-[4-bromo-n-butyl  88460 195194-80-0 50
                ]-
                Vinyl lauryl ether                71313 000765-14-0 50

36  25.052  0.02 C:\Database\NIST11.L
                3-Eicosene, (E)-                 127771 074685-33-9 90
                Octacosane                       209565 000630-02-4 83
                1-Docosene                       151537 001599-67-3 78

37  25.355  0.12 C:\Database\NIST11.L
                Octadecane, 1-chloro-            134595 003386-33-2 89
                Octadecane                       105884 000593-45-3 83
```

```
                2-Piperidinone, N-[4-bromo-n-butyl  88460 195194-80-0 64
                ]-

38  25.741  0.03 C:\Database\NIST11.L
                Octadecane                         105886 000593-45-3 60
                17-Pentatriacontene                234187 006971-40-0 53
                2-Piperidinone, N-[4-bromo-n-butyl  88460 195194-80-0 50
                ]-

39  26.112  0.07 C:\Database\NIST11.L
                Octadecane                         105886 000593-45-3 86
                Eicosane                           129490 000112-95-8 86
                Hentriacontane                     223851 000630-04-6 83
```

Thu Sep 26 19:18:53 2019

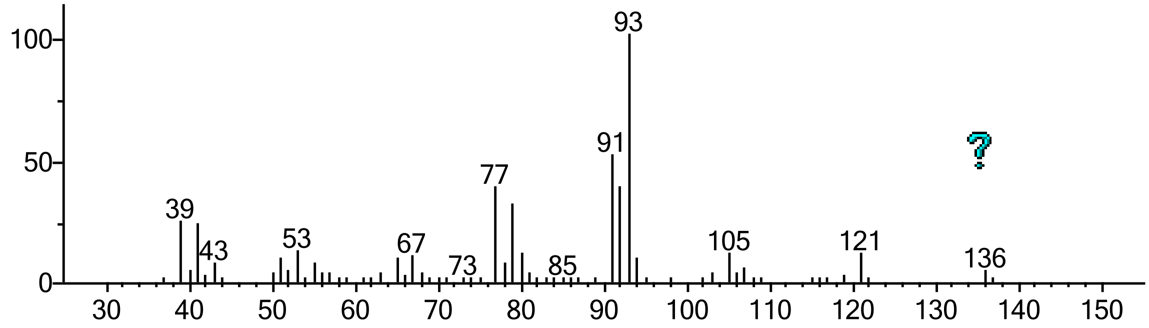

(Text File) Scan 18 (3.224 min): 0102020.D\ data.ms

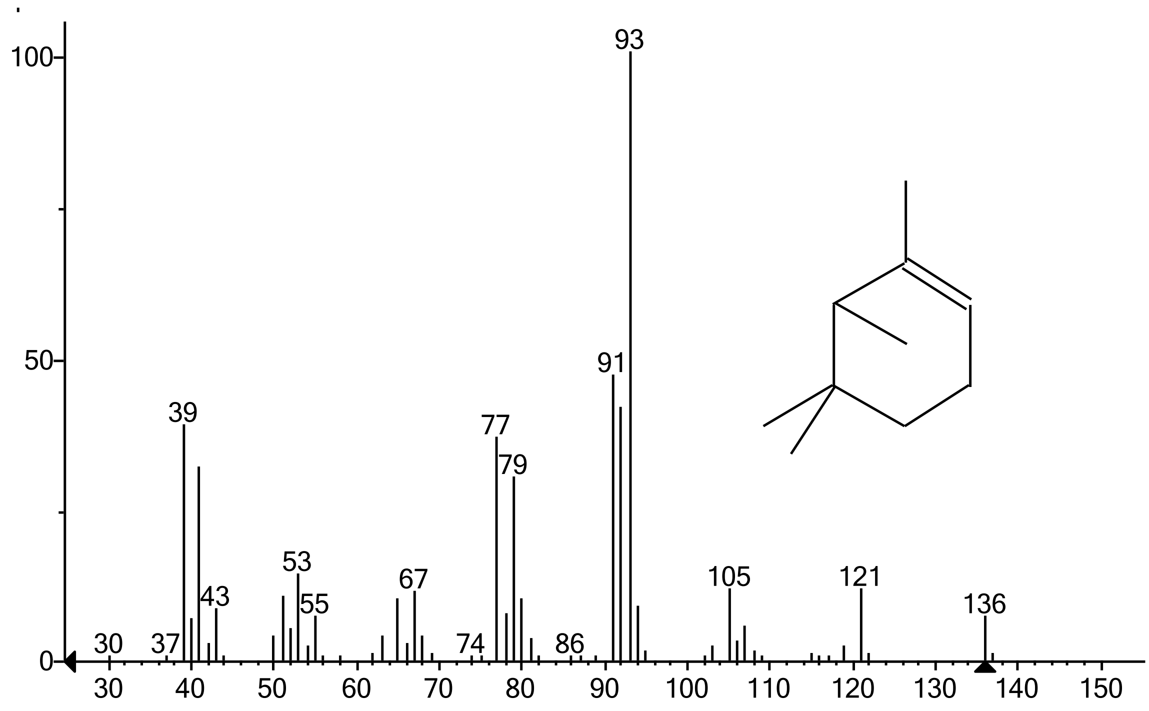

(mainlib) α-Pinene

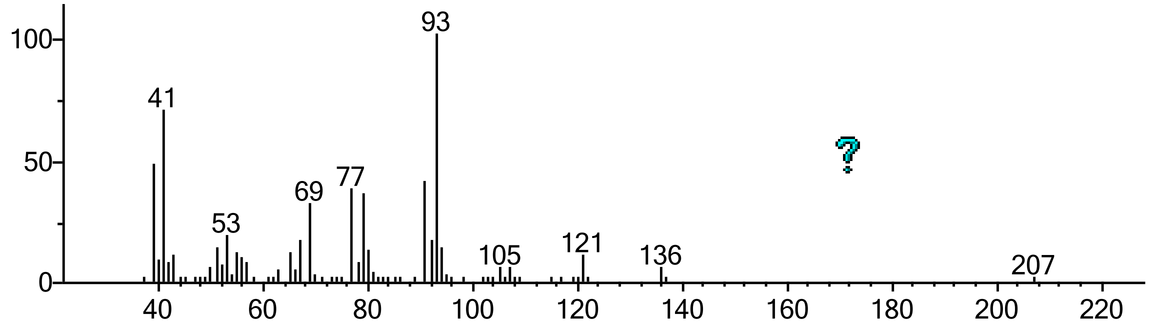

(Text File) Scan 78 (3.678 min): 0102020.D\ data.ms

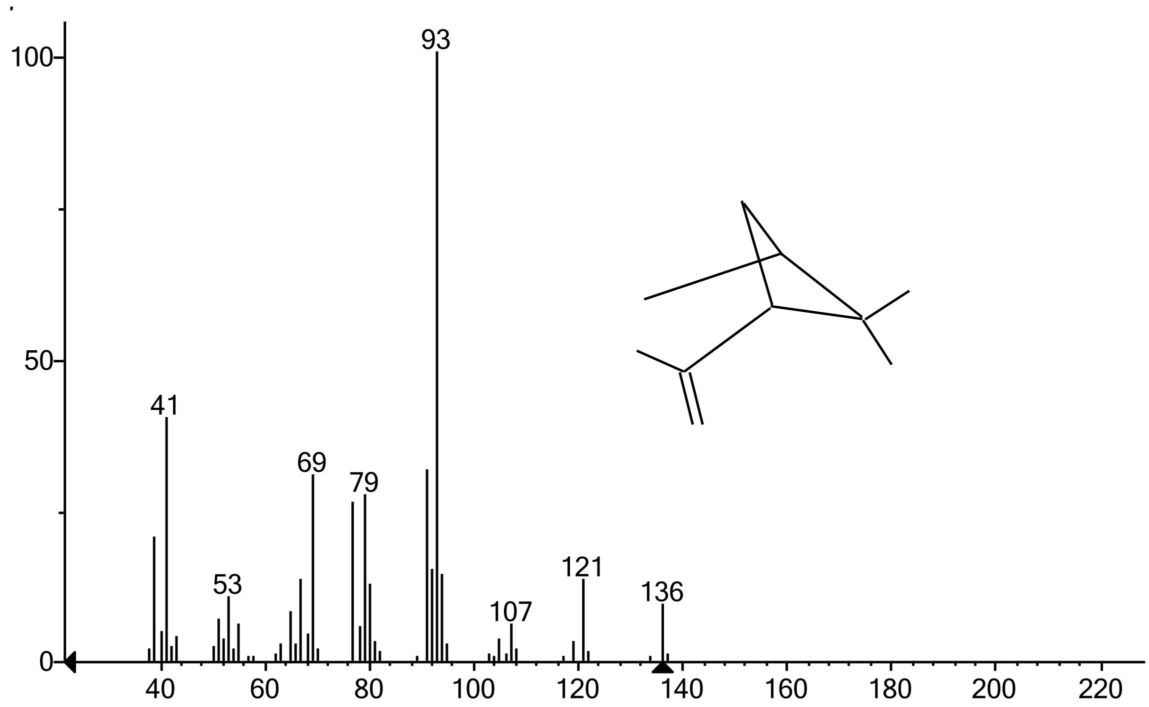

(replib) Bicyclo[3.1.1]heptane, 6,6-dimethyl-2-methylene-, (1S)-

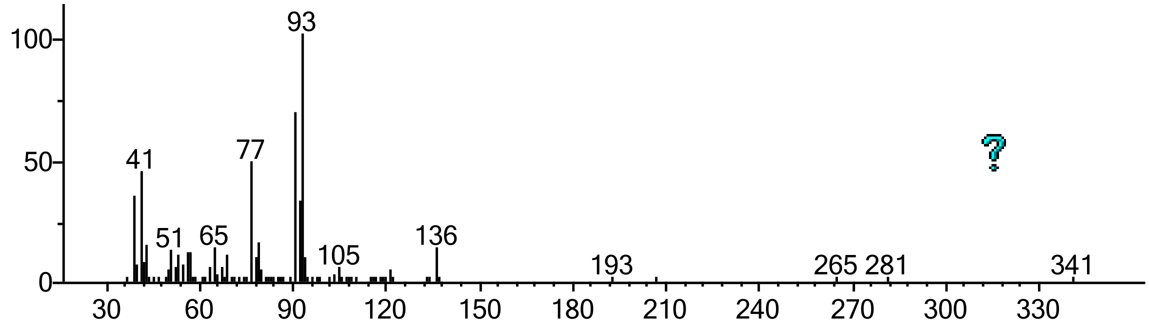

(Text File) Scan 146 (4.193 min): 0102020.D\ data.ms

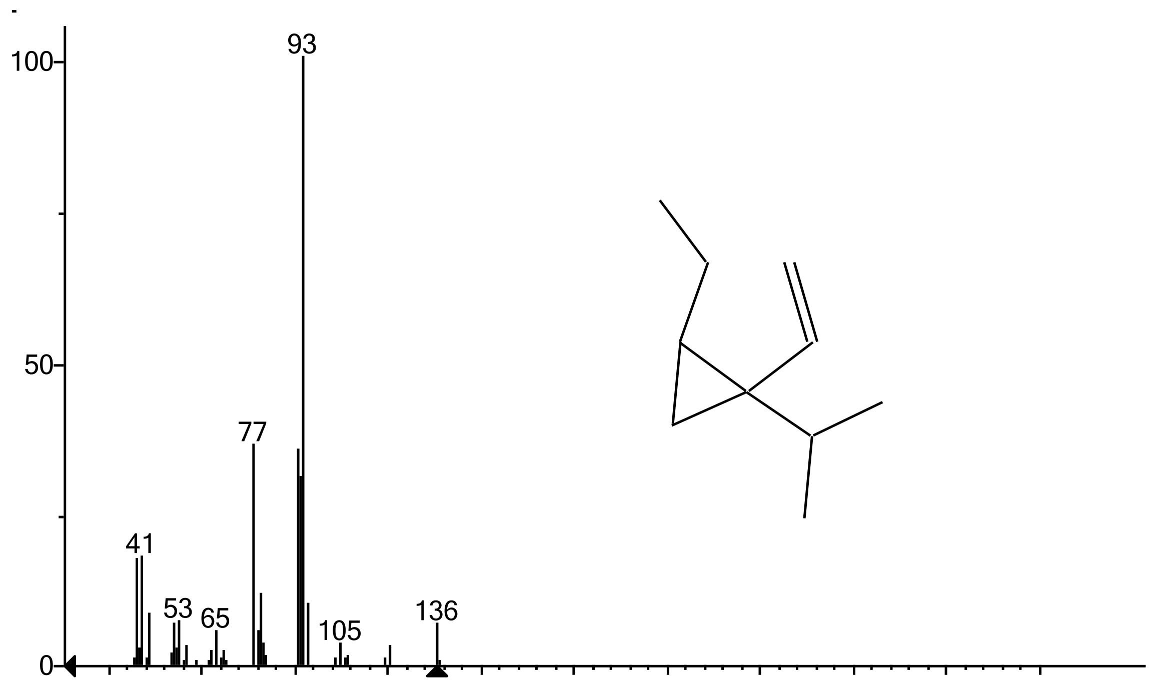

(mainlib) Bicyclo[3.1.0]hex-2-ene, 4-methyl-1-(1-methylethyl)-

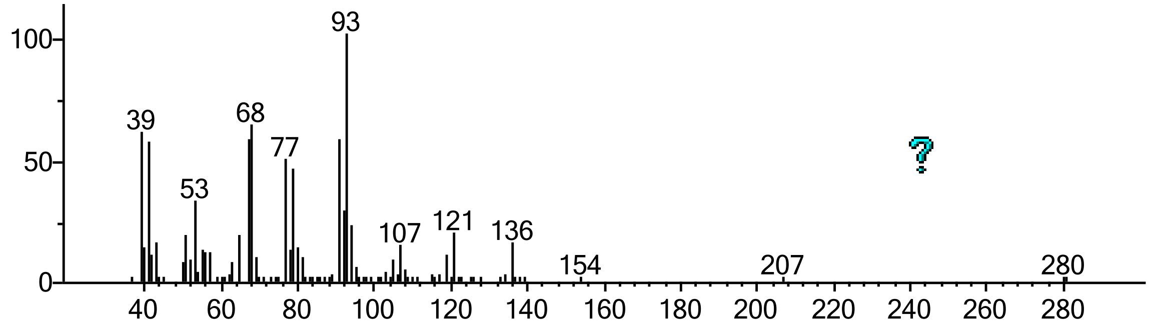

(Text File) Scan 188 (4.511 min): 0102020.D\ data.ms

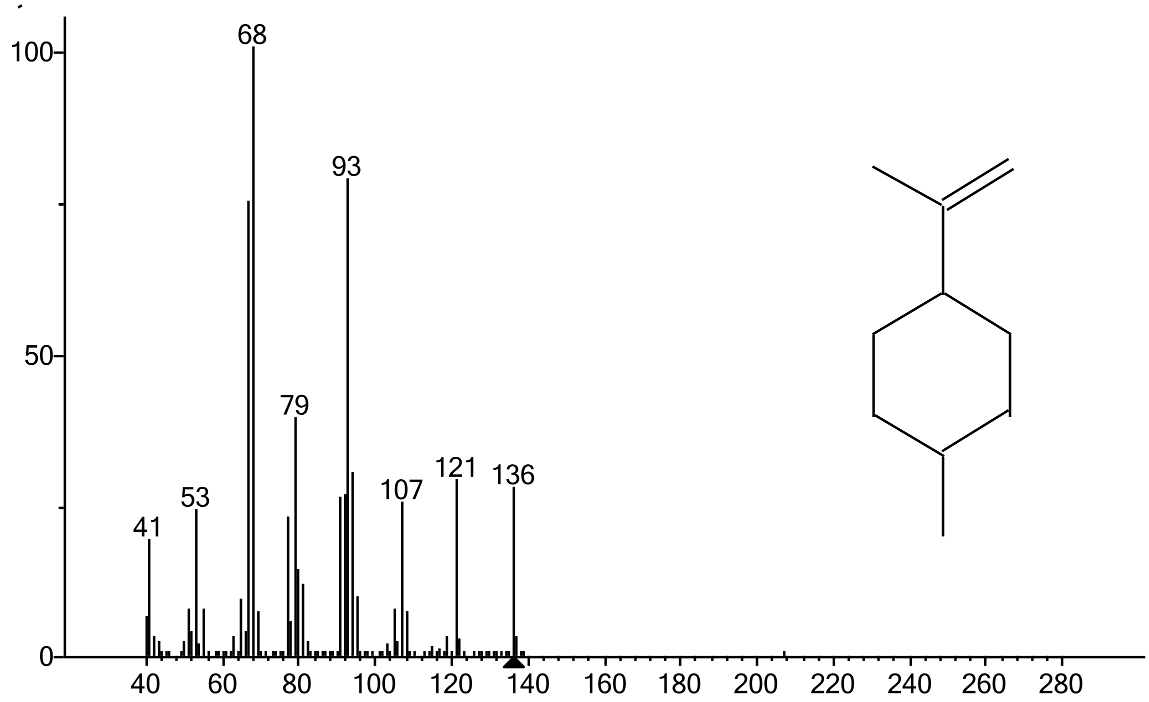

(replib) D-Limonene

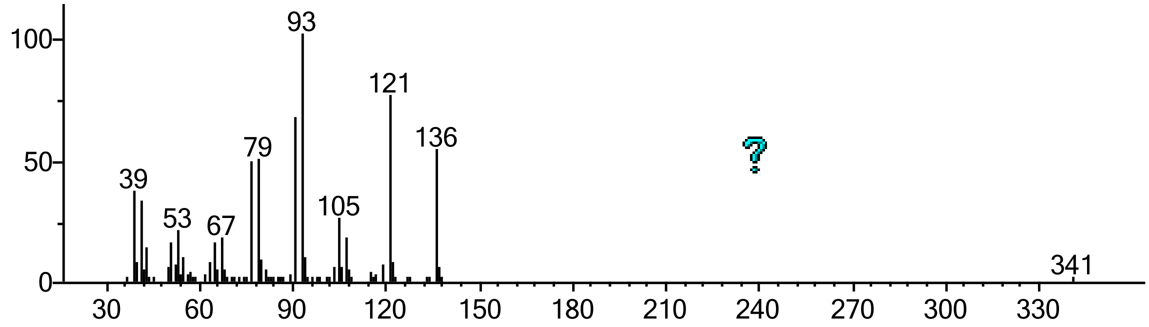

(Text File) Scan 292 (5.298 min): 0102020.D\ data.ms

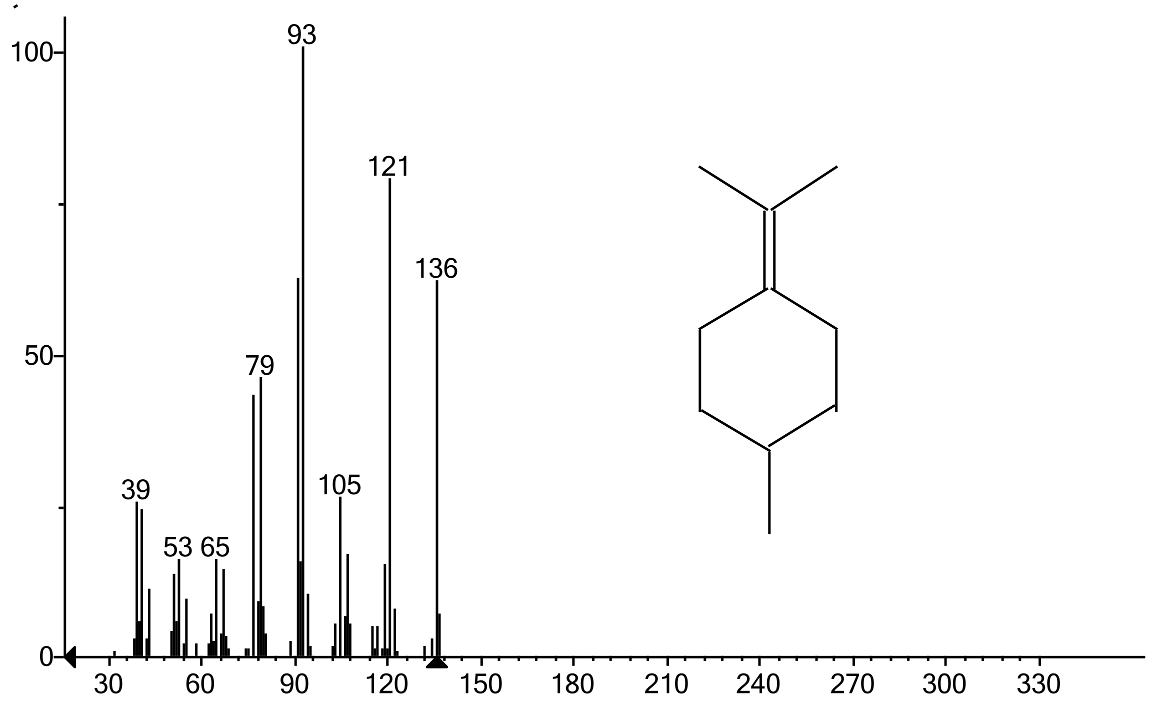

(mainlib) Cyclohexene, 1-methyl-4-(1-methylethylidene)-

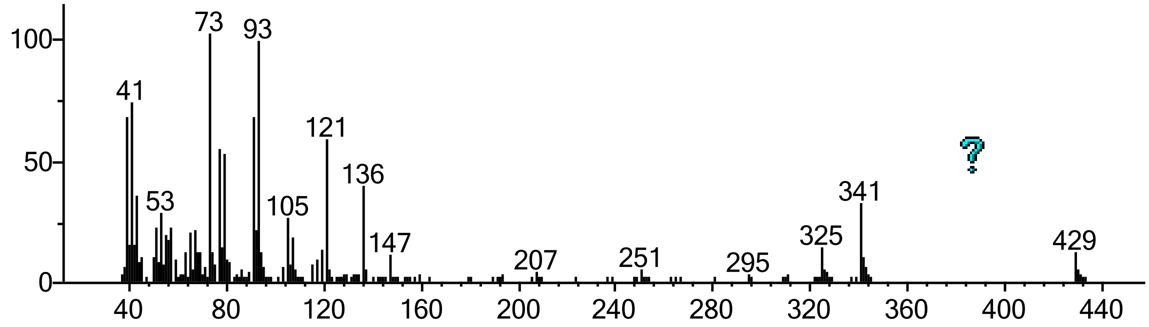

(Text File) Scan 665 (8.121 min): 0102020.D\ data.ms

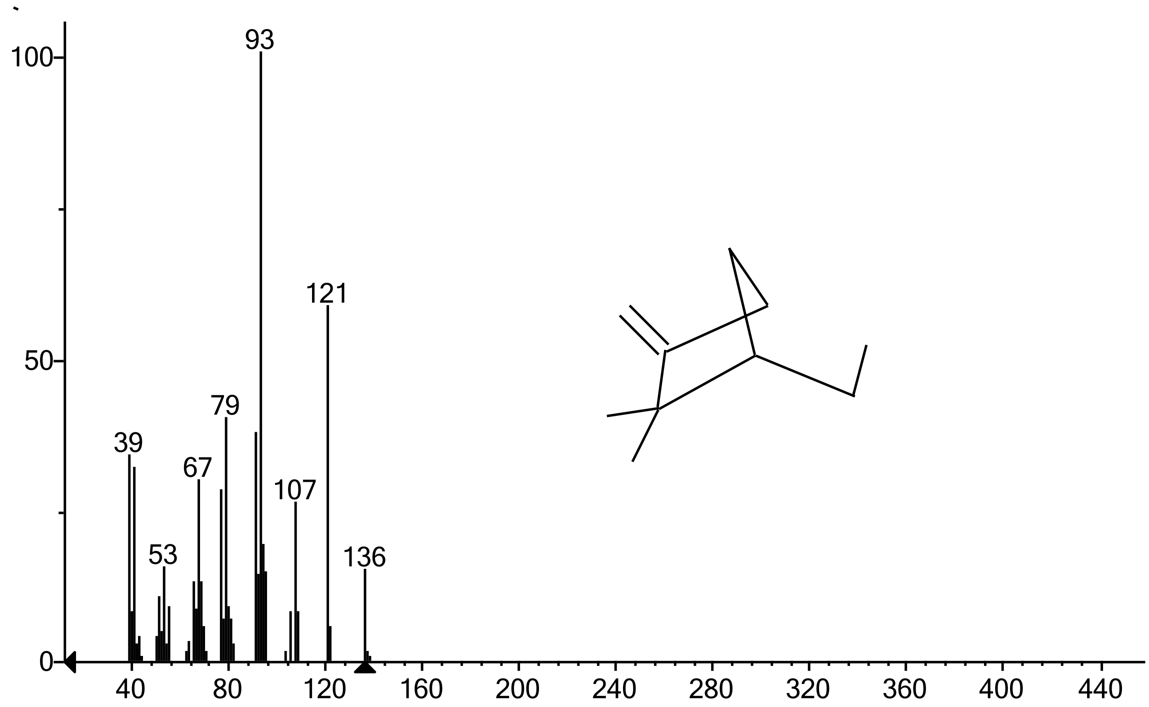

(mainlib) Camphene

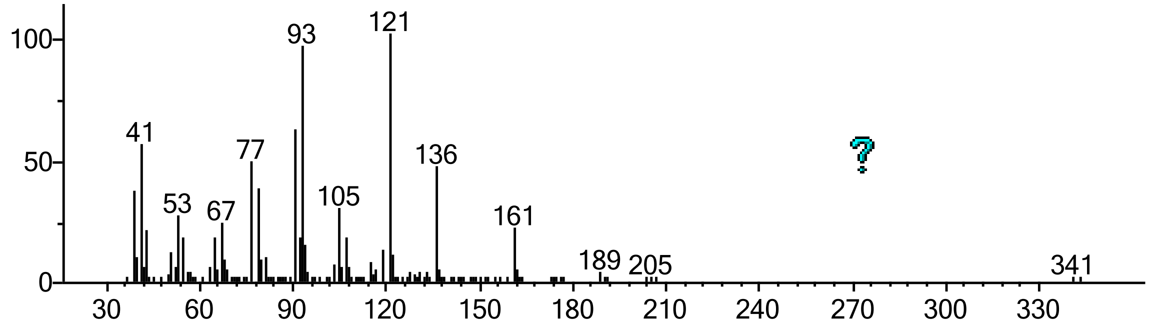

(Text File) Scan 705 (8.424 min): 0102020.D\ data.ms

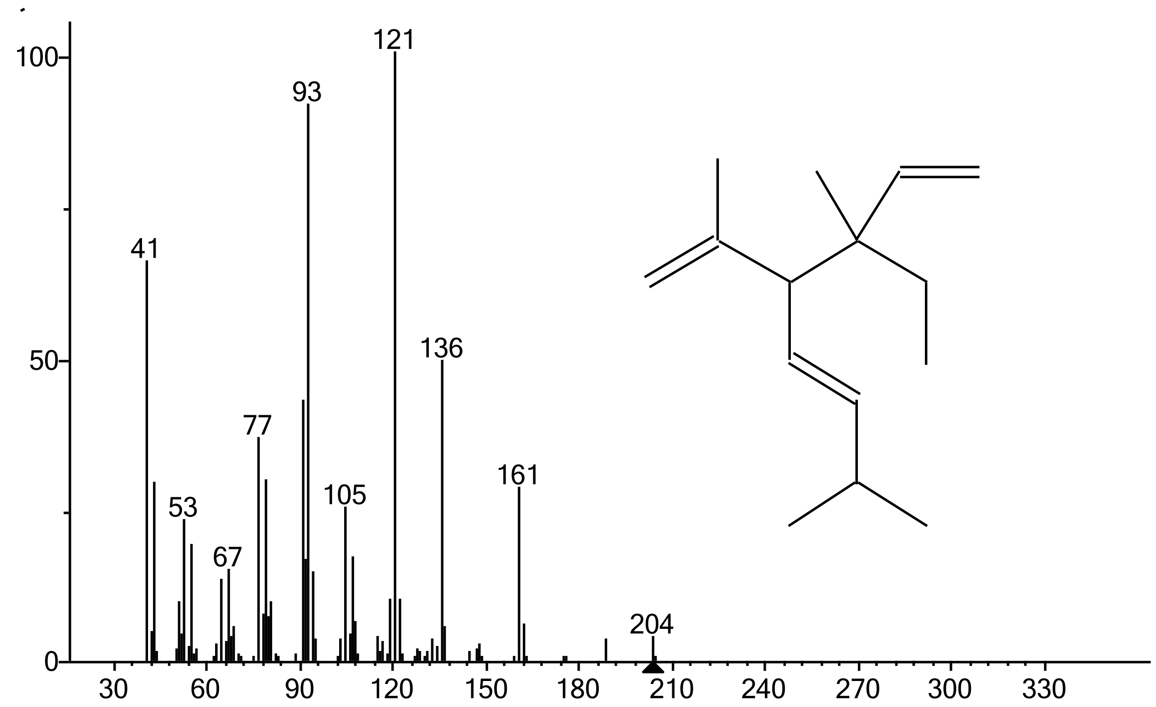

(replib) Cyclohexene, 4-ethenyl-4-methyl-3-(1-methylethenyl)-1-(1-methylethyl)-, (3R-trans)-

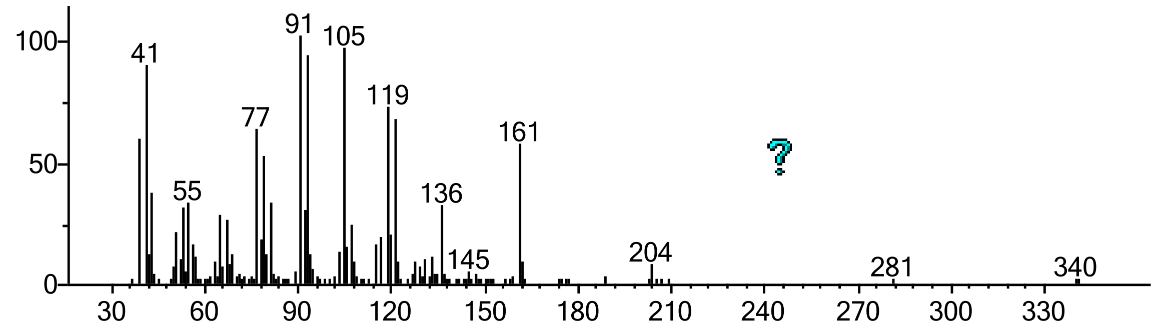

(Text File) Scan 726 (8.583 min): 0102020.D\ data.ms

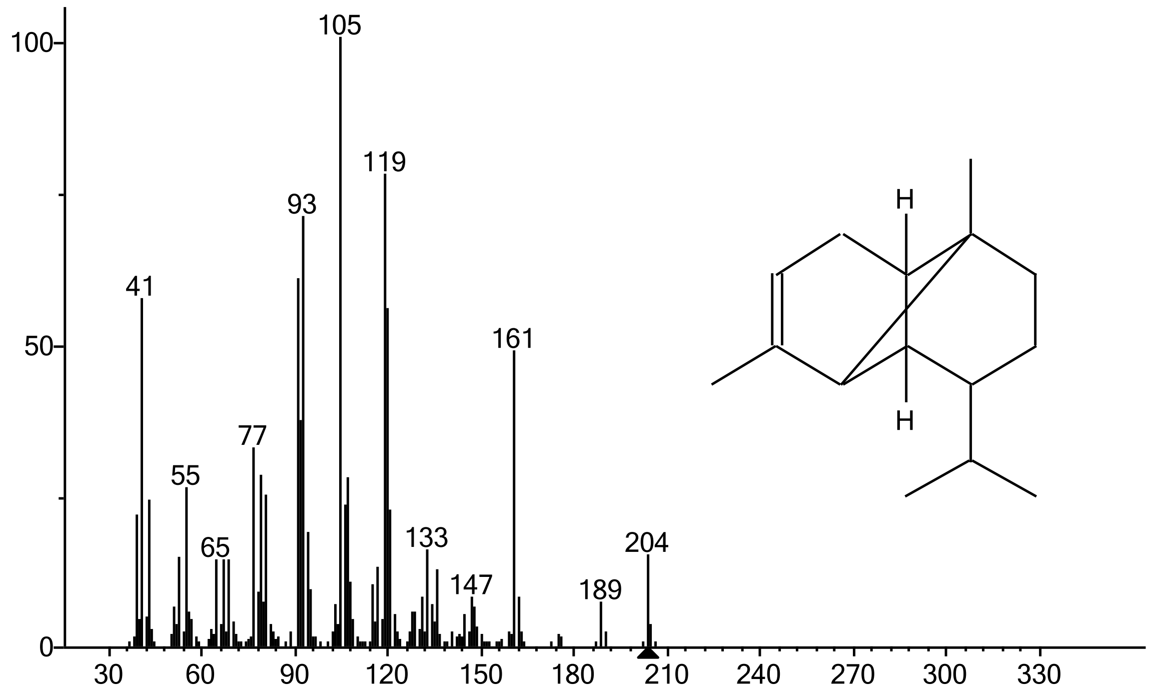

(mainlib) α-ylangene

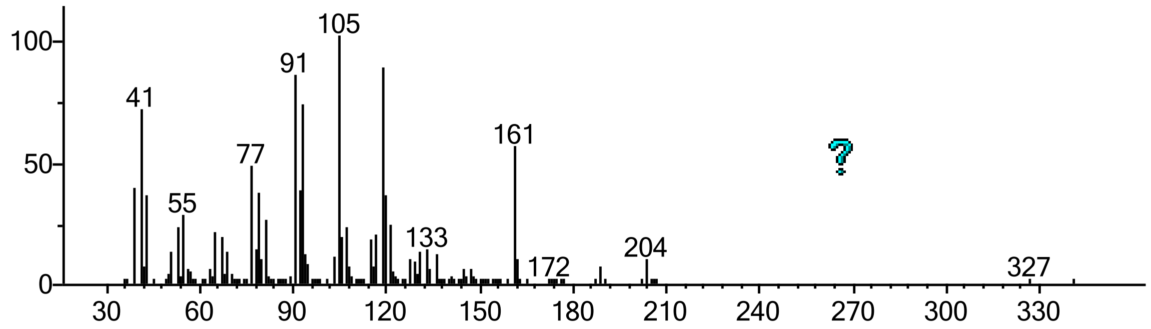

(Text File) Scan 772 (8.931 min): 0102020.D\ data.ms

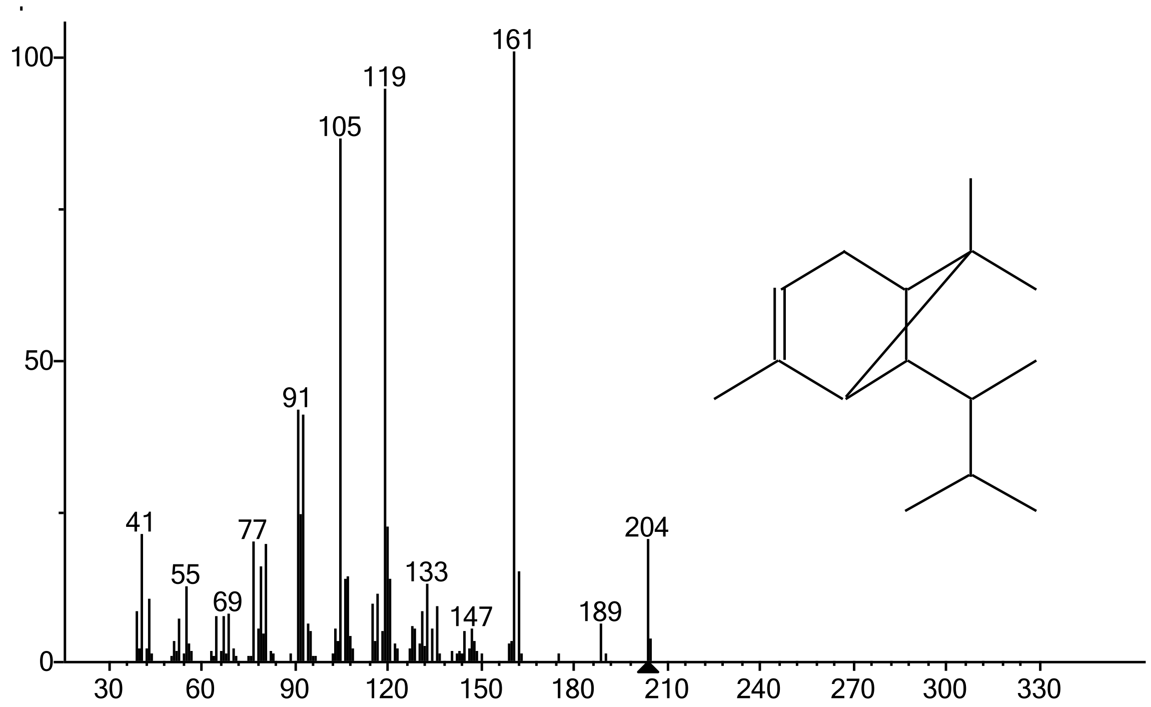

(mainlib) .alfa.-Copaene

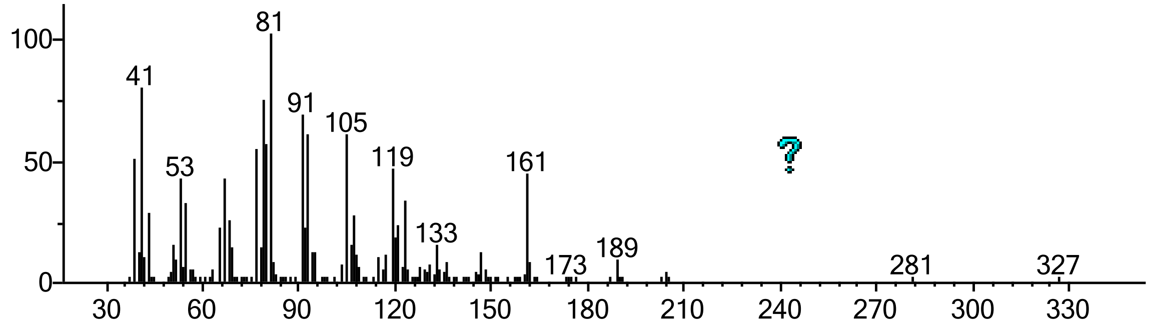

(Text File) Scan 799 (9.135 min): 0102020.D\ data.ms

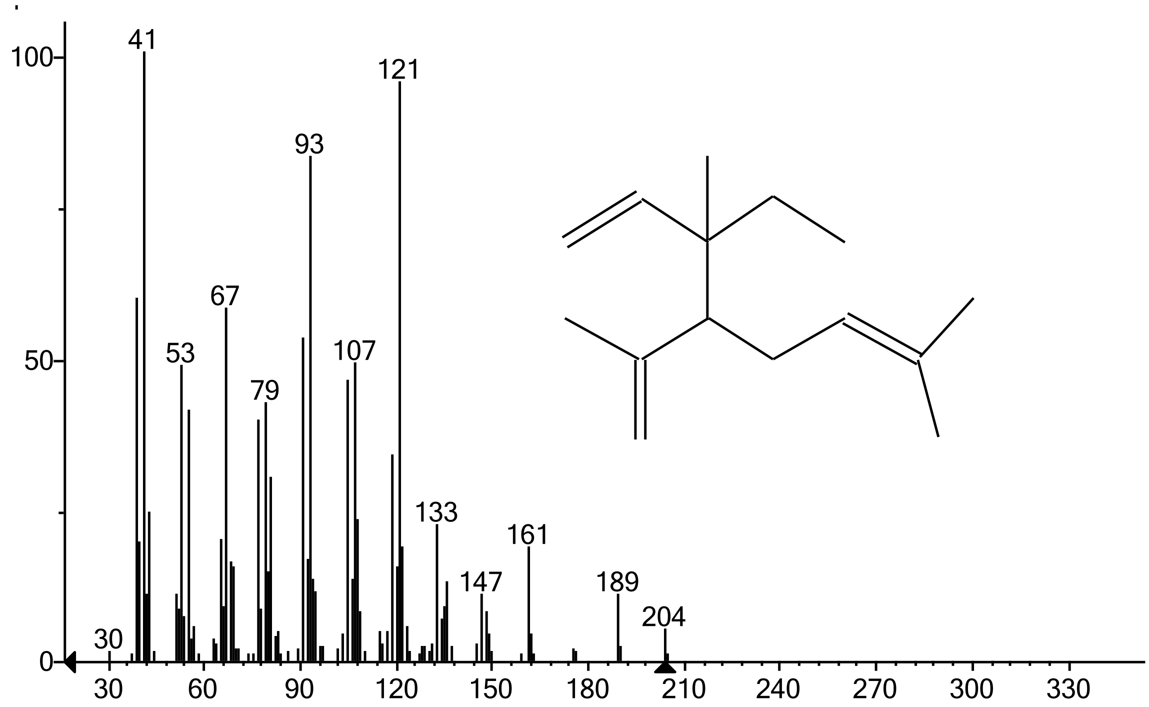

(replib) γ-Elemene

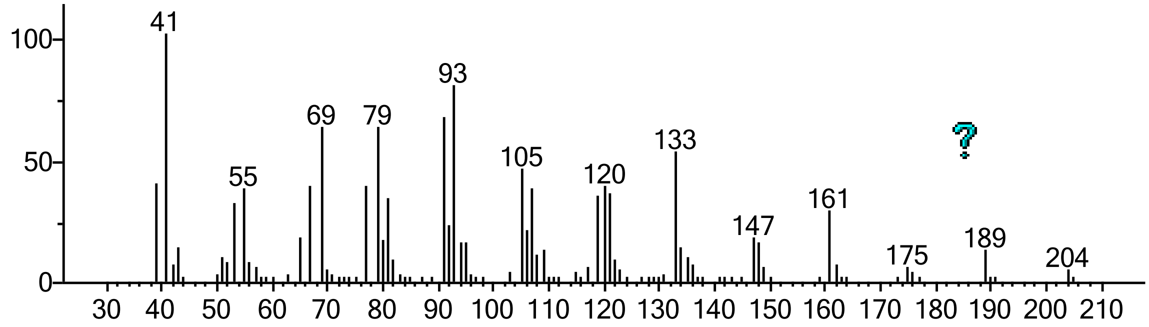

(Text File) Scan 885 (9.786 min): 0102020.D\ data.ms

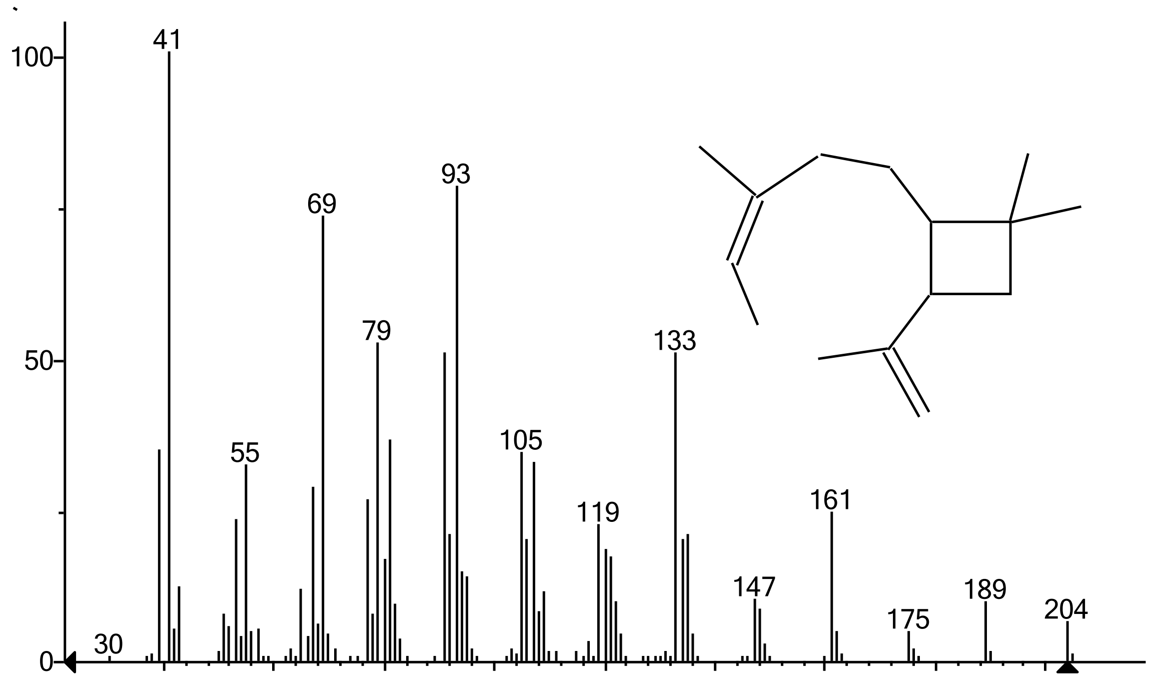

(mainlib) Bicyclo[7.2.0]undec-4-ene, 4,11,11-trimethyl-8-methylene-,[1R-(1R*,4Z,9S*)]-

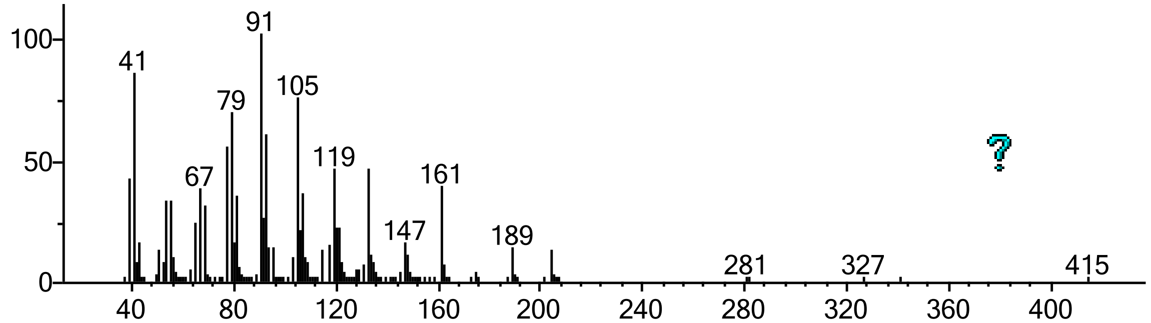

(Text File) Scan 905 (9.937 min): 0102020.D\ data.ms

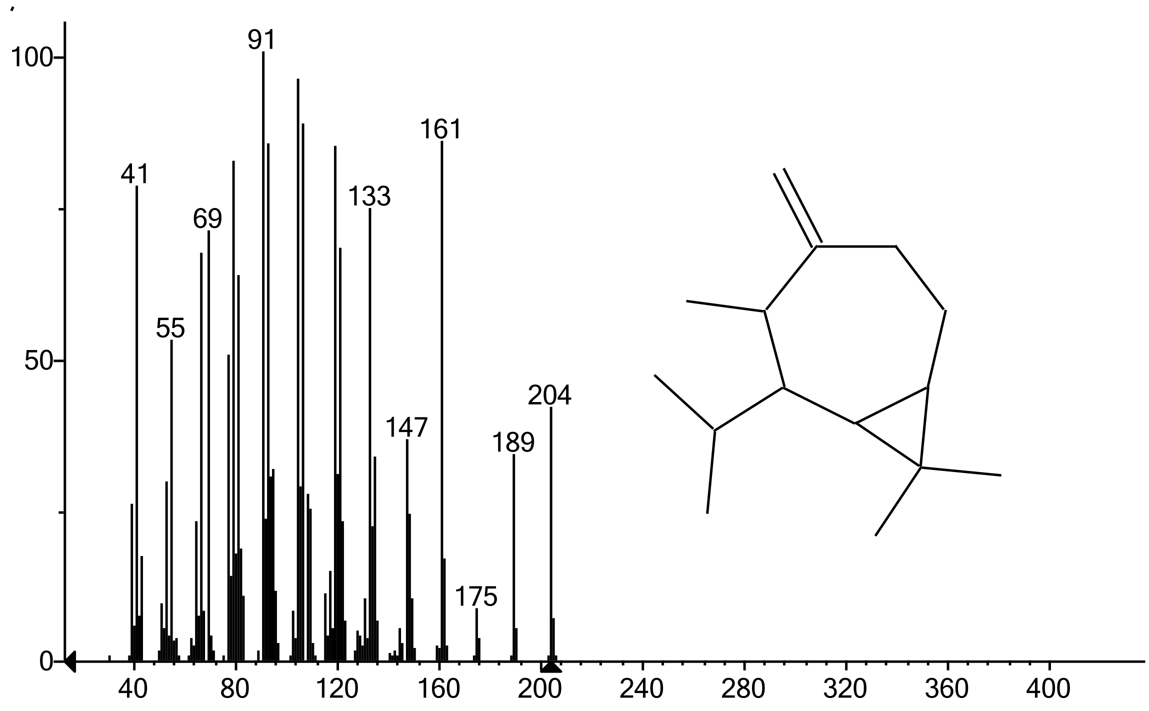

(replib) Aromandendrene

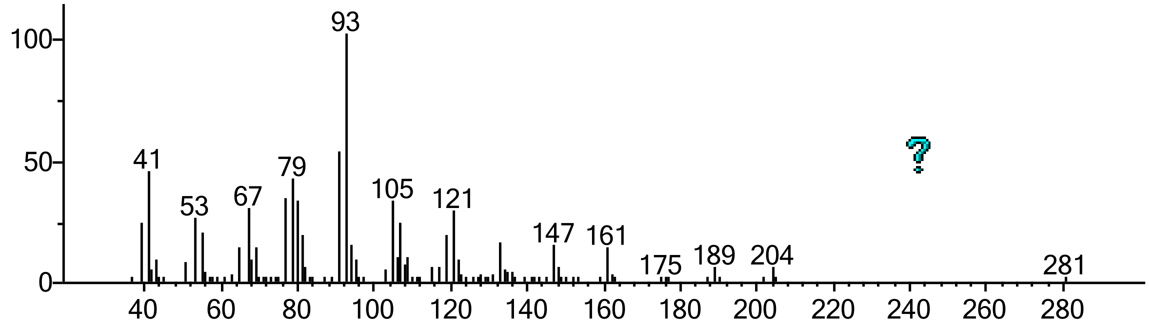

(Text File) Scan 916 (10.021 min): 0102020.D\ data.ms

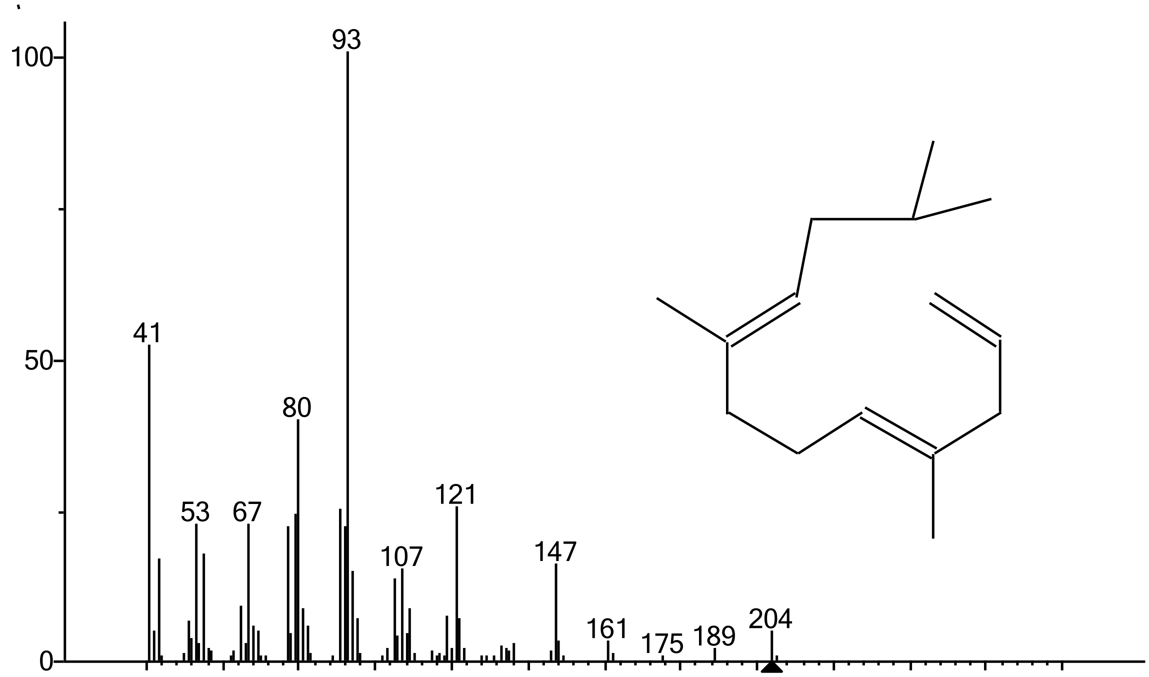

(replib) Humulene

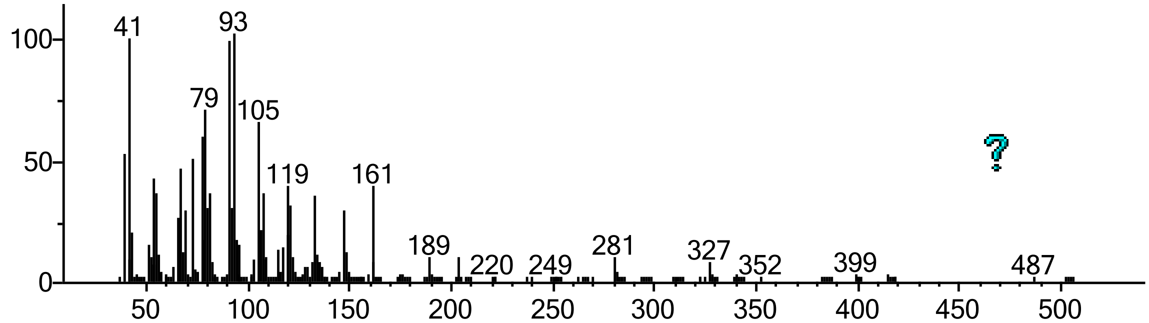

(Text File) Scan 946 (10.248 min): 0102020.D\ data.ms

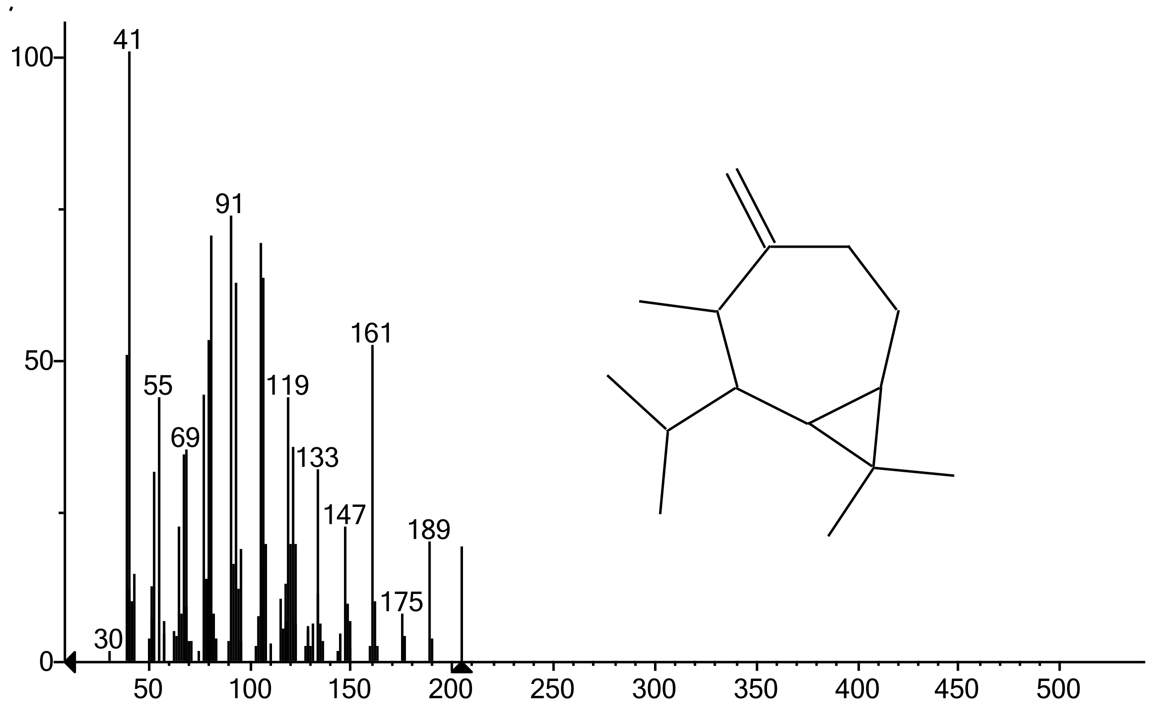

(replib) Alloaromadendrene

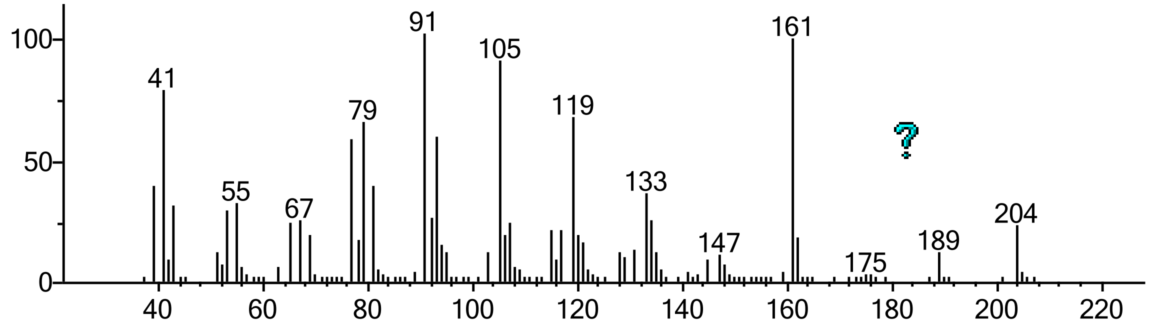

(Text File) Scan 1028 (10.868 min): 0102020.D\ data.ms

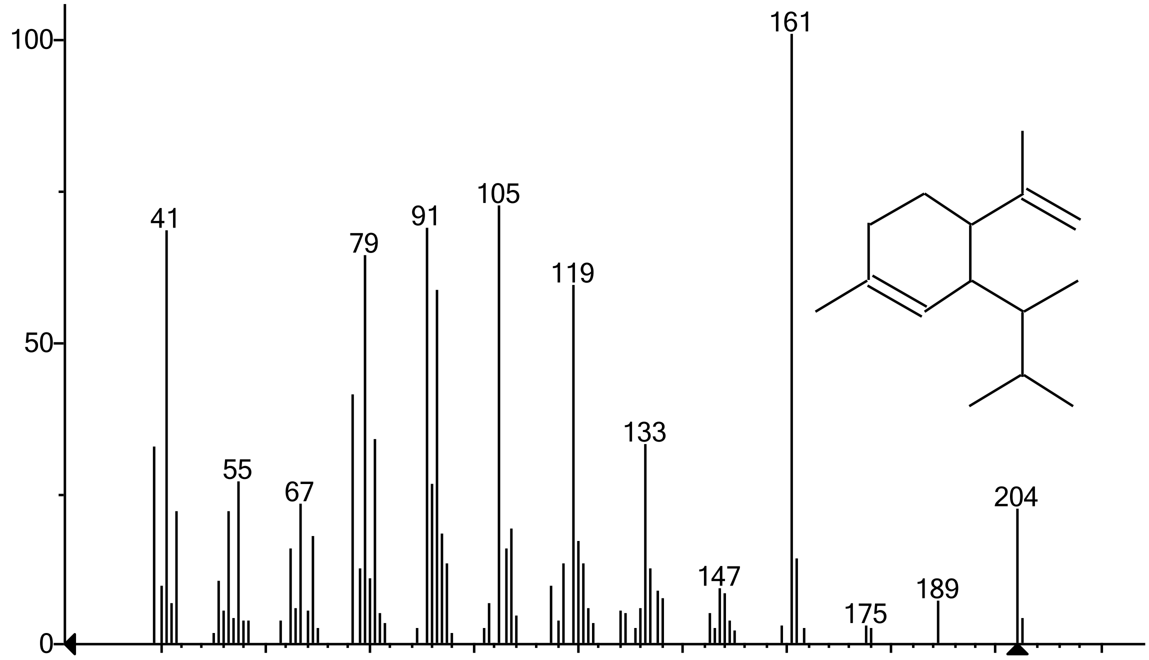

(replib) Naphthalene, 1,2,4a,5,6,8a-hexahydro-4,7-dimethyl-1-(1-methylethyl)-

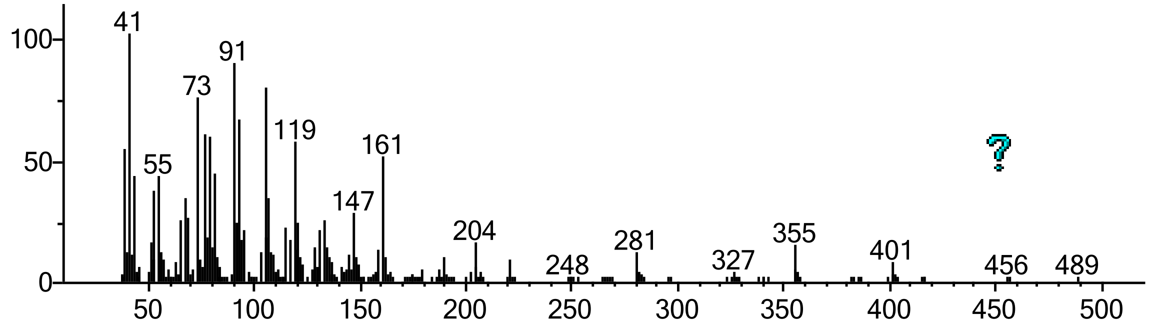

(Text File) Scan 1209 (12.238 min): 0102020.D\ data.ms

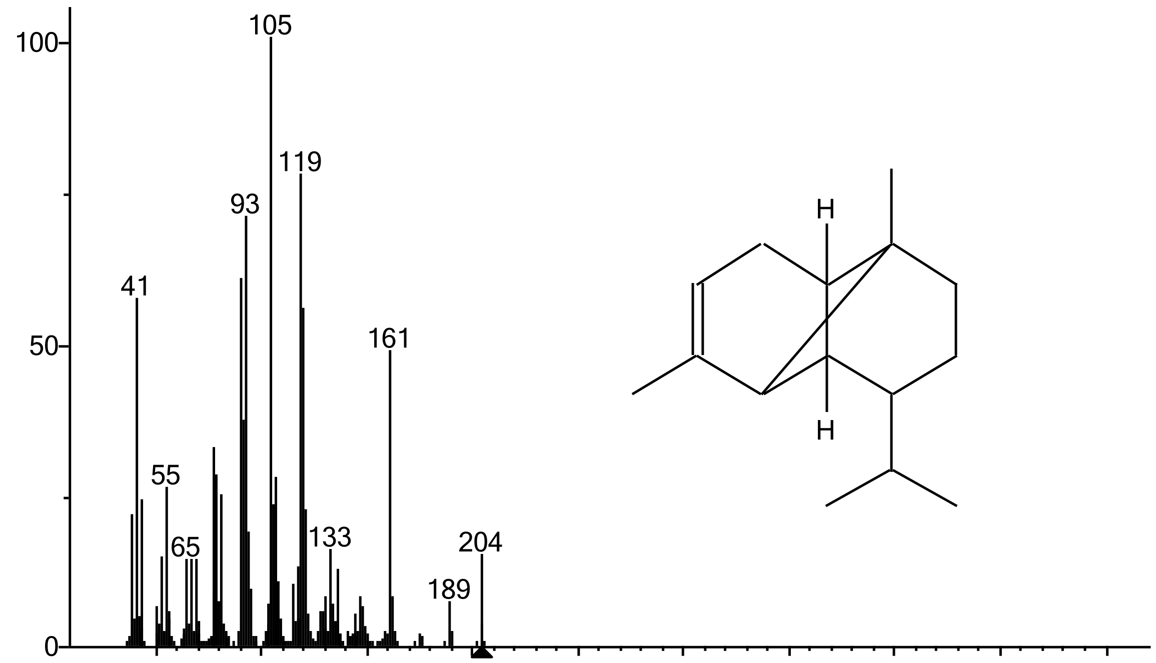

(mainlib) α-ylangene

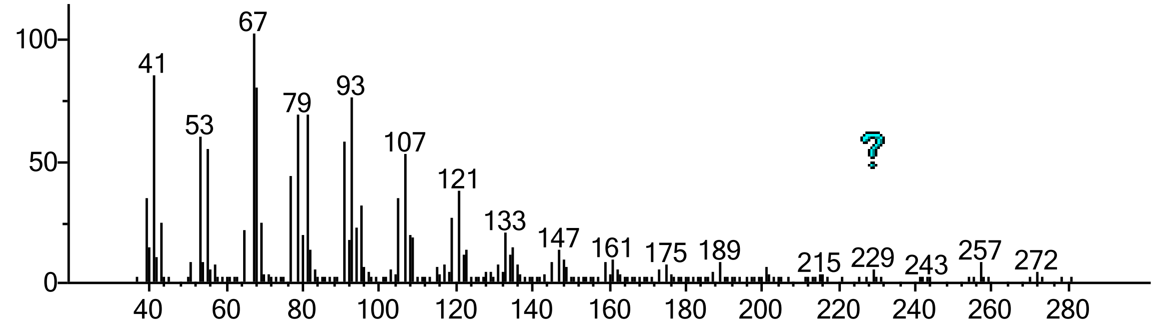

(Text File) Scan 1666 (15.697 min): 0102020.D\ data.ms

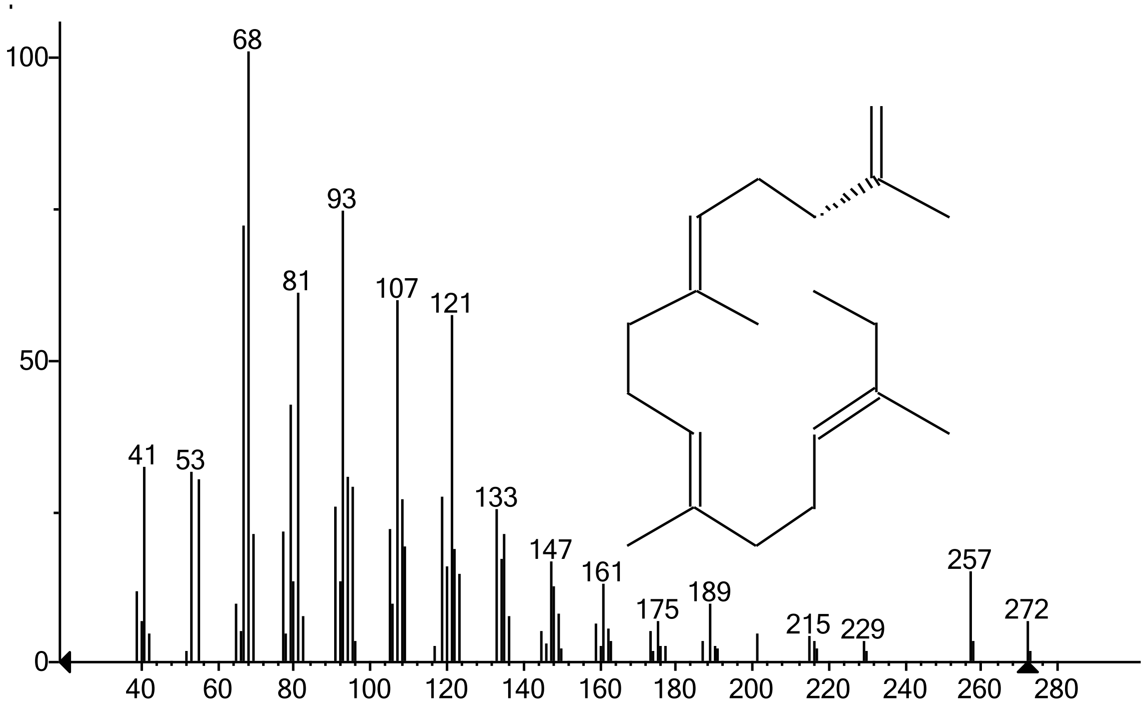

(mainlib) (R,1E,5E,9E)-1,5,9-Trimethyl-12-(prop-1-en-2-yl)cyclotetradeca-1,5,9-triene

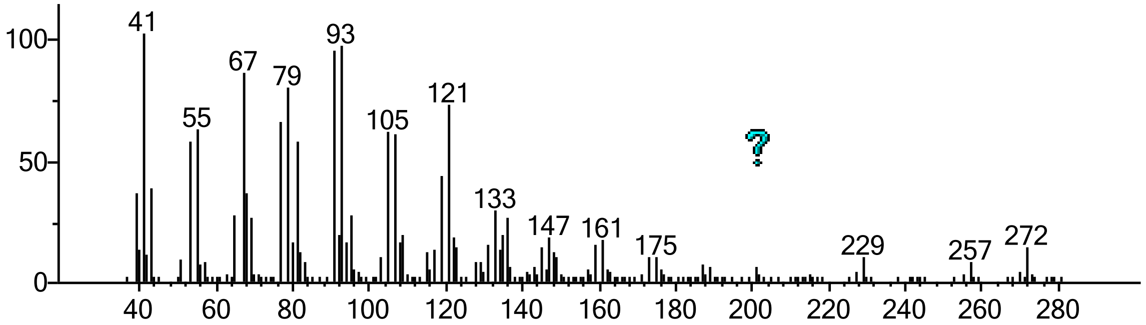

(Text File) Scan 1753 (16.356 min): 0102020.D\ data.ms

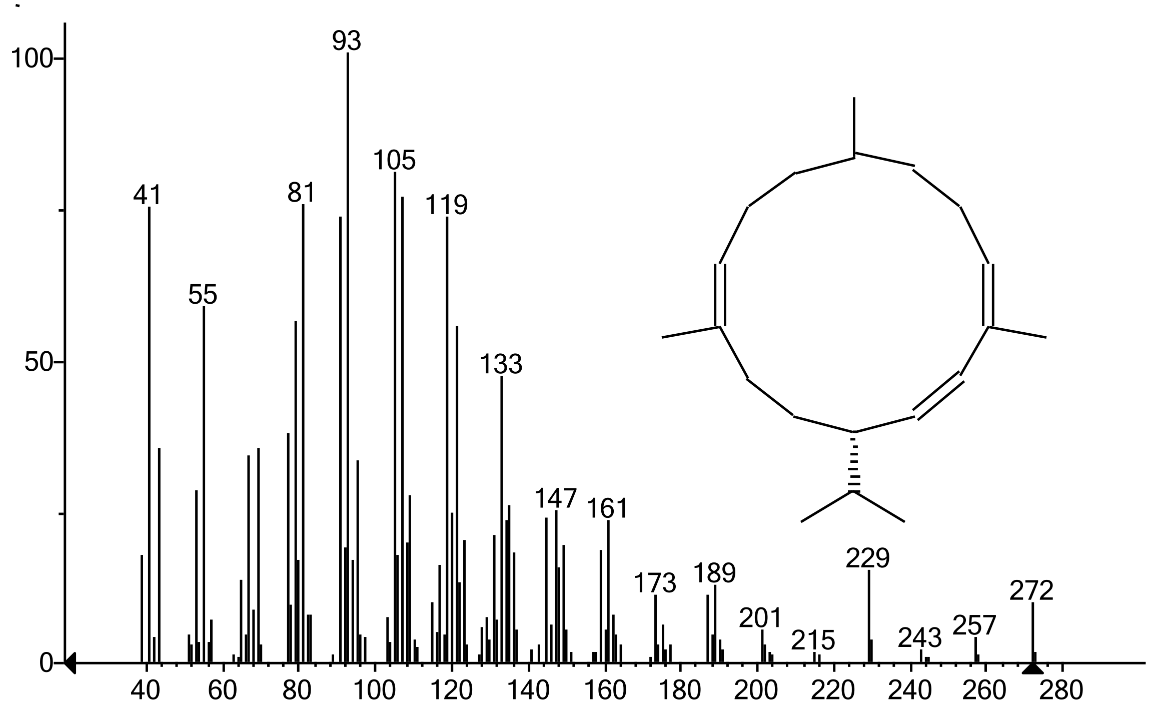

(replib) 1,3,6,10-Cyclotetradecatetraene, 3,7,11-trimethyl-14-(1-methylethyl)-, [S-(E,Z,E,E)]-

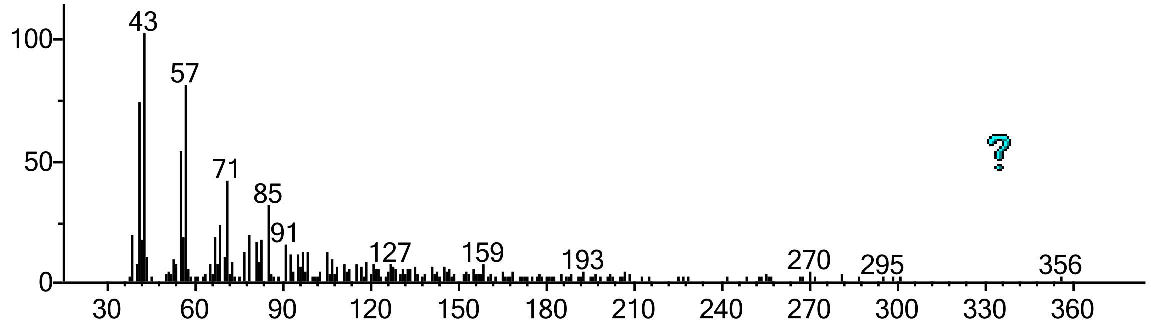

(Text File) Scan 2228 (19.951 min): 0102020.D\ data.ms

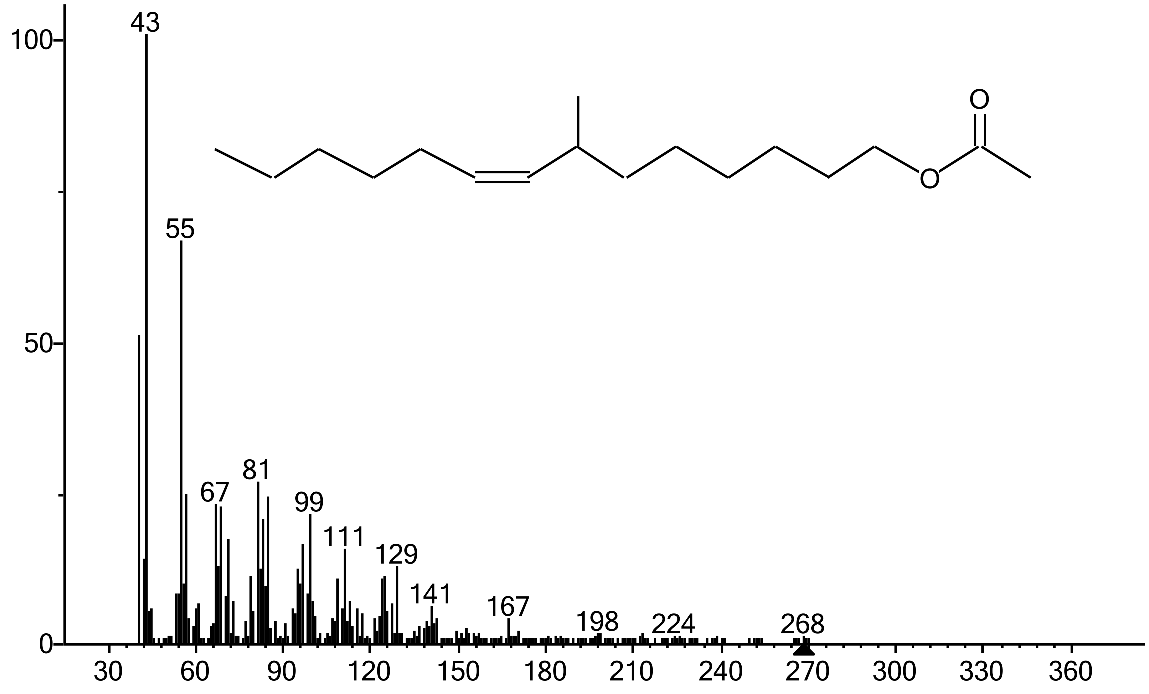

(mainlib) 7-Methyl-Z-tetradecen-1-ol acetate

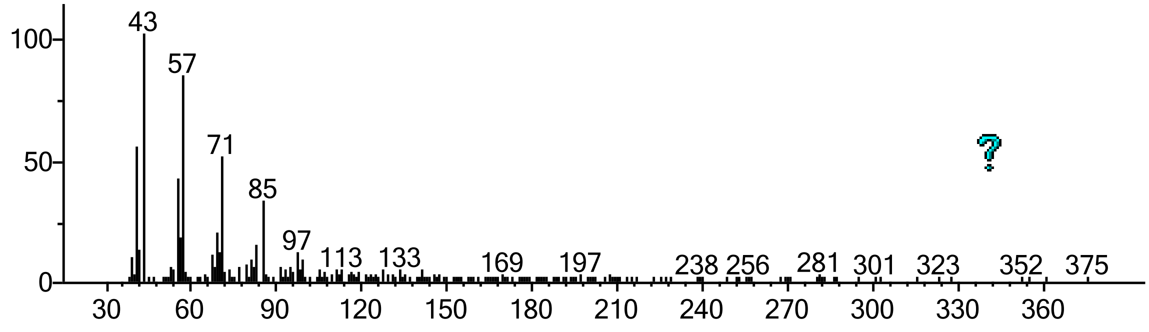

(Text File) Scan 2324 (20.677 min): 0102020.D\ data.ms

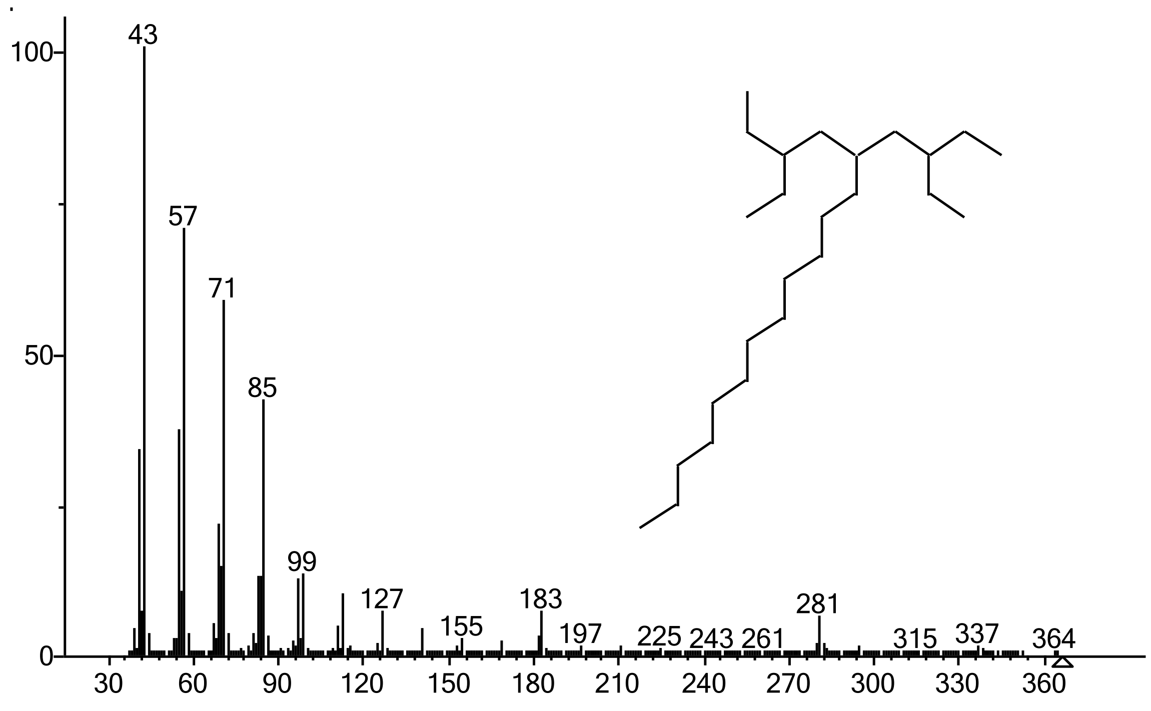

(mainlib) Octadecane, 3-ethyl-5-(2-ethylbutyl)-

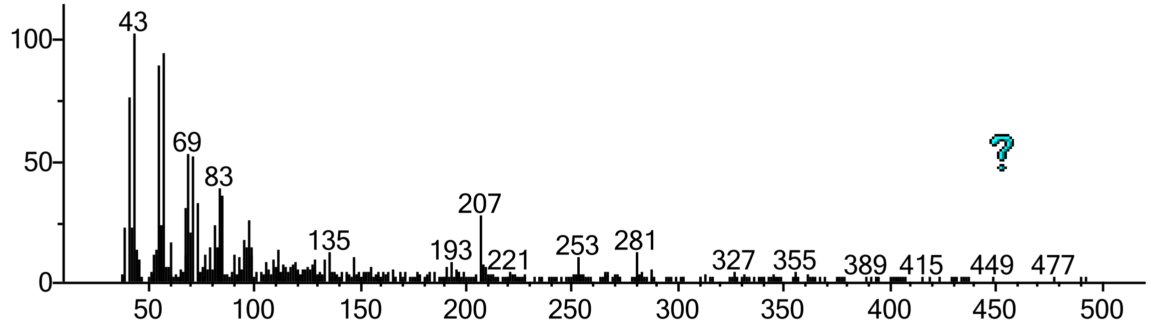

(Text File) Scan 3042 (26.112 min): 0102020.D\ data.ms

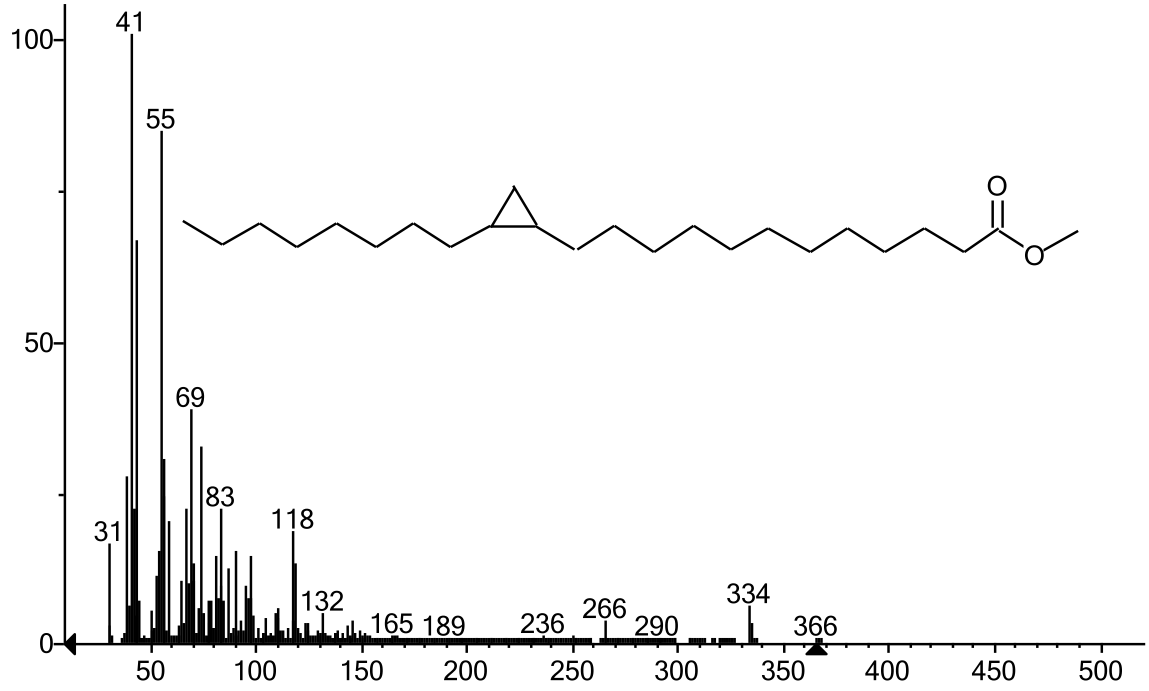

(mainlib) Cyclopropanedodecanoic acid, 2-octyl-, methyl ester

INSTRUMENT CONTROL PARAMETERS: GC-MSD

C:\MSDCHEM\1\METHODS\Plant ext .M

Wed Sep 25 18:02:35 2019

Control Information

------- -----------

Sample Inlet   : GC

Injection Source : GC ALS

Mass Spectrometer : Enabled

No Sample Prep method has been assigned to this method.

Oven

Equilibration Time   8 min

Max Temperature	310 degrees C
Slow Fan	Disabled
Oven Program	On
60 °C for 0.5 min	
then 10 °C/min to 300 °C for 3 min	
Run Time	27.5 min

Front Injector

Syringe Size	10 µL
Injection Volume	0.2 µL
Injection Repetitions	1
Injection Delay	0 sec
Solvent A Washes (PreInj)	1
Solvent A Washes (PostInj)	2
Solvent A Volume	8 µL
Solvent B Washes (PreInj)	1
Solvent B Washes (PostInj)	1
Solvent B Volume	8 µL
Sample Washes	2
Sample Wash Volume	5 µL
Sample Pumps	1
Dwell Time (PreInj)	0 min
Dwell Time (PostInj)	0 min
Solvent Wash Draw Speed	300 µL/min
Solvent Wash Dispense Speed	6000 µL/min
Sample Wash Draw Speed	300 µL/min
Sample Wash Dispense Speed	6000 µL/min
Injection Dispense Speed	6000 µL/min
Viscosity Delay	7 sec
Sample Depth	Disabled

Sample Overlap

Sample overlap is not enabled

Front SS Inlet He

Mode	Split
Heater	On 270 °C
Pressure	On 10.983 psi
Total Flow	On 3.6407 mL/min
Septum Purge Flow	On 1 mL/min
Gas Saver	On 20 mL/min After 2 min
Split Ratio	1 :1
Split Flow	1.3203 mL/min

Thermal Aux 2 (MSD Transfer Line)

Heater	On
Temperature Program	On
300 °C for 0 min	
Run Time	27.5 min

Column #1

Agilent 19091S-433: 469.56509

HP-5MS 5% Phenyl Methyl Silox

325 °C: 30 m x 250 µm x 0.25 µm

In: Front SS Inlet He

Out: Vacuum

(Initial)	60 °C

Pressure	11.649 psi
Flow	1.3203 mL/min
Average Velocity	42.082 cm/sec
Holdup Time	1.1882 min
Flow Program	Off
1.3203 mL/min for 0 min	
Run Time	27.5 min

Signals

Signal #1: Test Plot	Save Off
	50 Hz
Signal #2: Test Plot	Save Off
	50 Hz
Signal #3: Test Plot	Save Off
	50 Hz
Signal #4: Test Plot	Save Off
	50 Hz

MS ACQUISITION PARAMETERS

General Information

------- -----------

Tune File : atune.u

Acquistion Mode : Scan/SIM

MS Information

-- -----------

```
Solvent Delay            : 3.00 min


EMV Mode                 : Gain Factor

Gain Factor              : 2.00

Resulting EM Voltage     : 2459

[Scan Parameters]


Low Mass                 : 35.0

High Mass                : 550.0

Threshold                : 150

Sample #                 : 2       A/D Samples    4

Plot 1 low mass          : 50.0

Plot 1 high mass         : 550.0

[Sim Parameters]


GROUP 1

Group ID                 : 1

Resolution               : Low

Plot 1 Ion               : 74.10

Ions/Dwell In Group      (  Mass,  Dwell)

                         ( 74.10,    100)


[MSZones]


MS Source                : 250 C   maximum 280 C

MS Quad                  : 150 C   maximum 200 C
```

END OF MS ACQUISITION PARAMETERS

TUNE PARAMETERS for SN: us73226898

Trace Ion Detection is OFF.

```
EMISSION    :     34.610
ENERGY      :     69.922
REPELLER    :     34.814
IONFOCUS    :     90.157
ENTRANCE_LE :      0.000
EMVOLTS     :   2988.235
                              Actual EMV  :    2458.82
                              GAIN FACTOR :       2.02
AMUGAIN     :   2066.000
AMUOFFSET   :    118.750
FILAMENT    :      1.000
DCPOLARITY  :      0.000
ENTLENSOFFS :     16.063
MASSGAIN    :   -504.000
MASSOFFSET  :    -40.000
```

END OF TUNE PARAMETERS

END OF INSTRUMENT CONTROL PARAMETERS
