# Supplementary material for: β‐Caryophyllene‐Rich Mercurialis perennis Leaf Essential Oil: GC–MS Profiling, Antioxidant Activity, Molecular Docking, and Molecular Dynamics Analysis
Source: Chem Biodivers. 2026 May 15;23:e71337. doi: 10.1002/cbdv.71337 (PMC13178399; doi:10.1002/cbdv.71337)
Supplement: Supplementary file 2 — Supporting File 2: cbdv71337‐sup‐0002‐SuppMat.pdf [file CBDV-23-e71337-s002.pdf]

Protein	ɣ-Muurolene	
	**2D**	**3D**
1oag	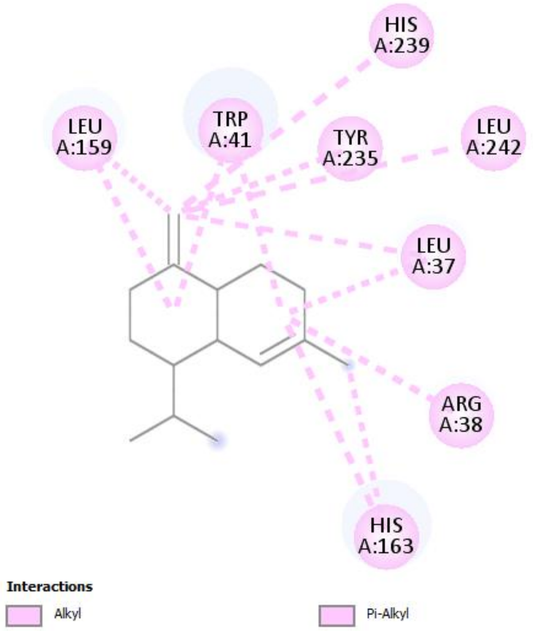	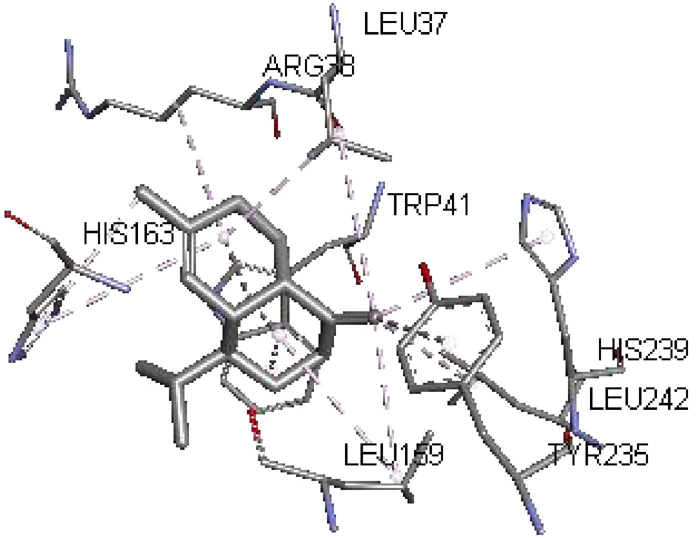

6xv4

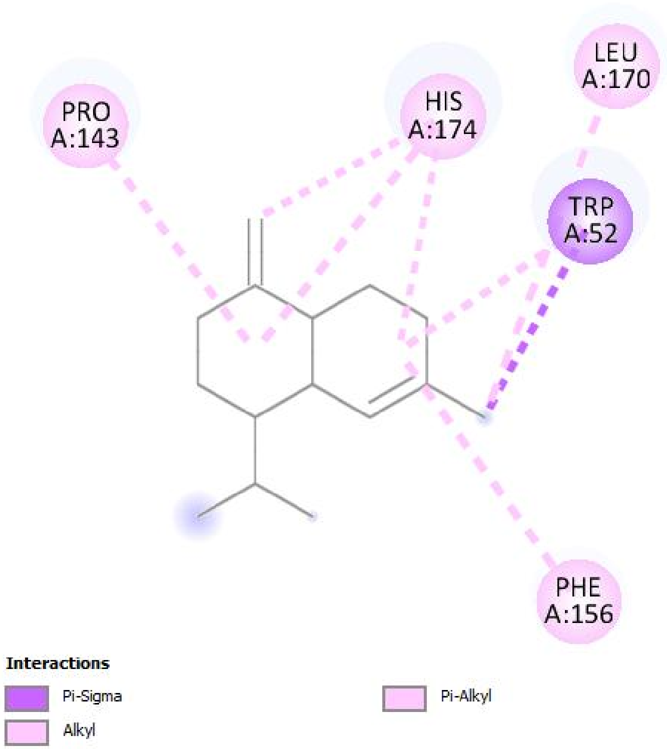
PRO
A:143
HIS
A:174
LEU
A:170
TRP
A:52
PHE
A:156
Interactions
Pi-Sigma
Alkyl
Pi-Alkyl

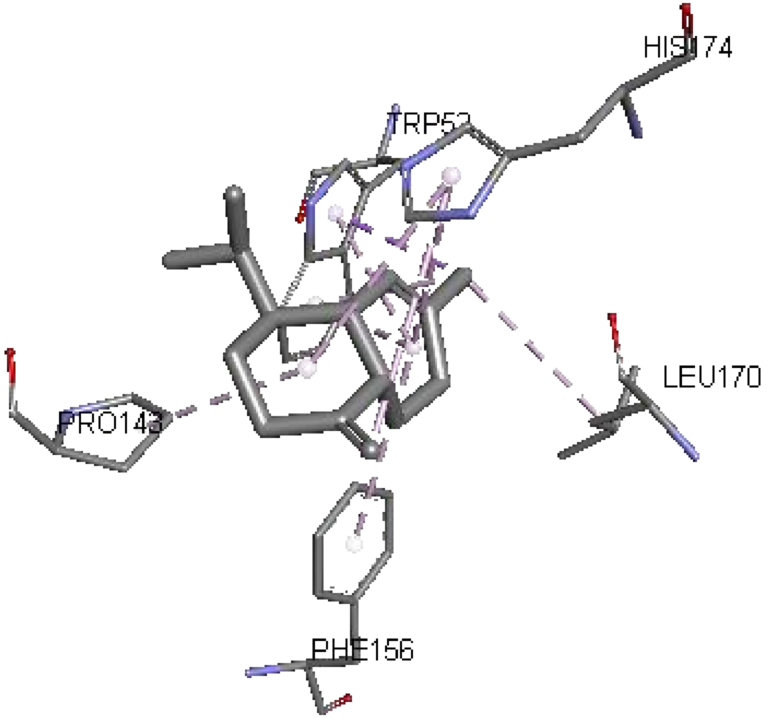
HIS174
TRP52
LEU170
PRO143
PHE156

9h1m

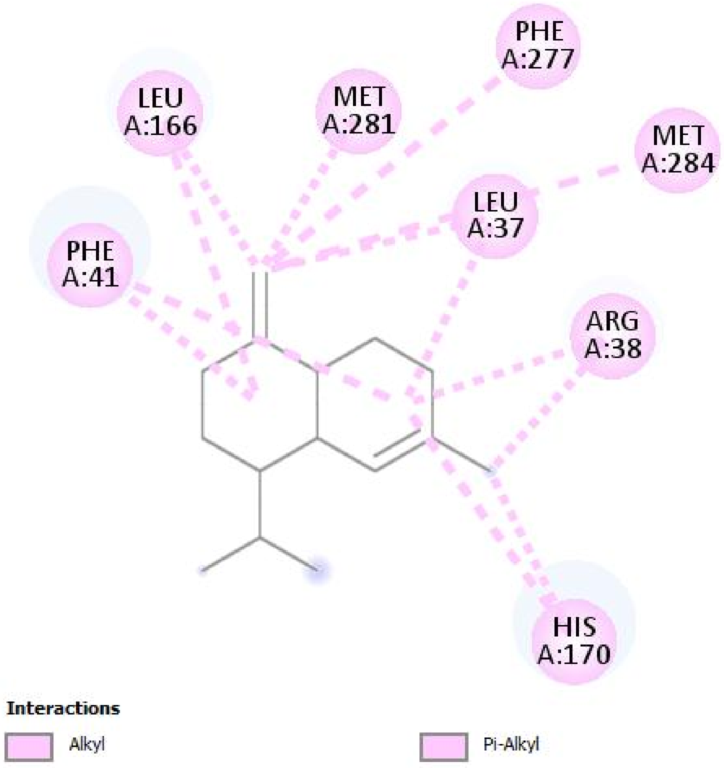

PHE A:277
LEU A:166
MET A:281
MET A:284
LEU A:37
PHE A:41
ARG A:38
HIS A:170
Interactions
Alkyl
Pi-Alkyl

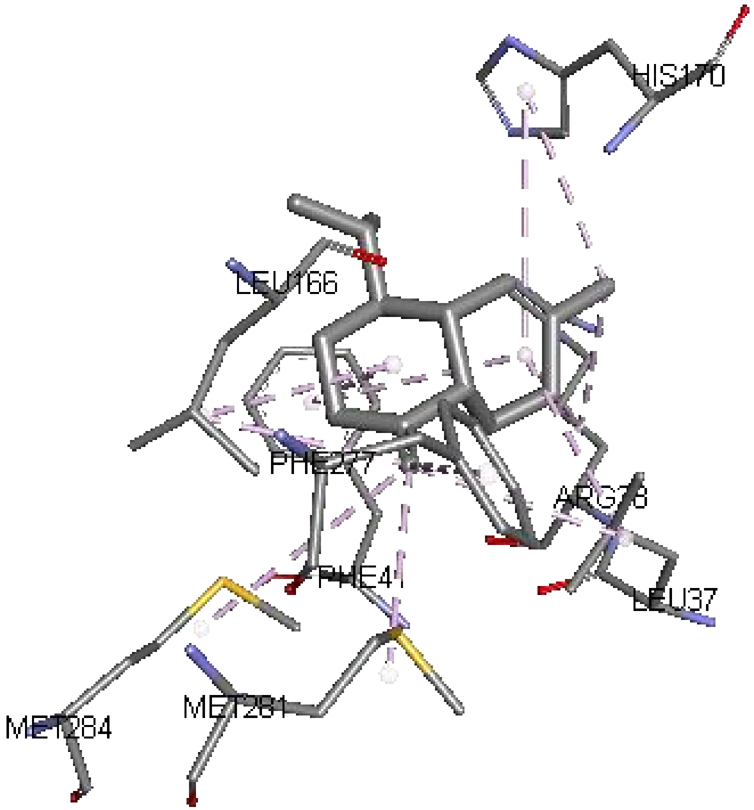

HIS170
LEU166
PHE277
ARG38
PHE41
LEU37
MET284
MET281

Protein	Caryophyllene	
	**2D**	**3D**
1oag	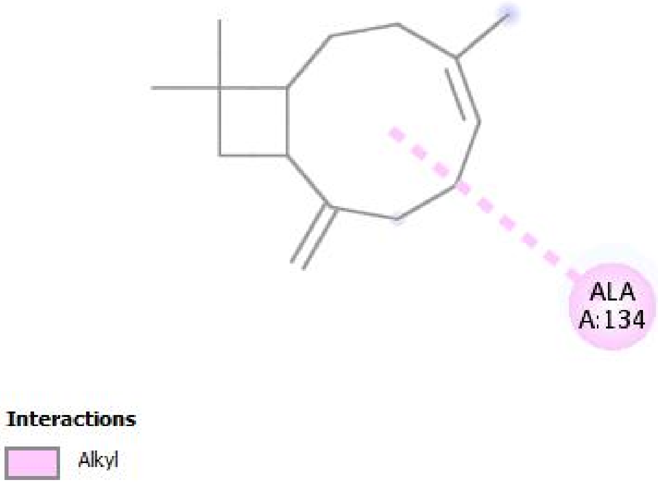	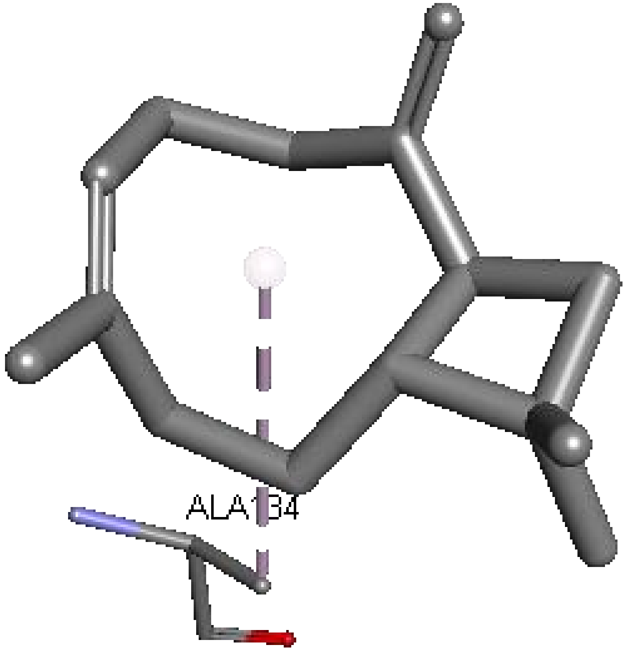

6xv4

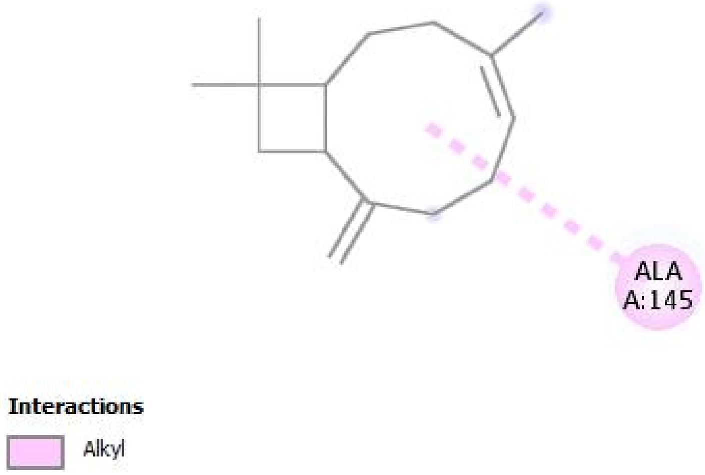

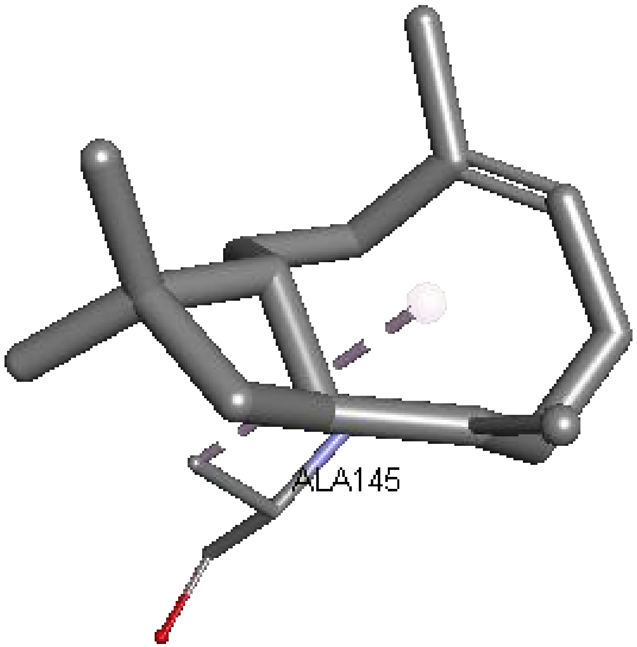

9h1m

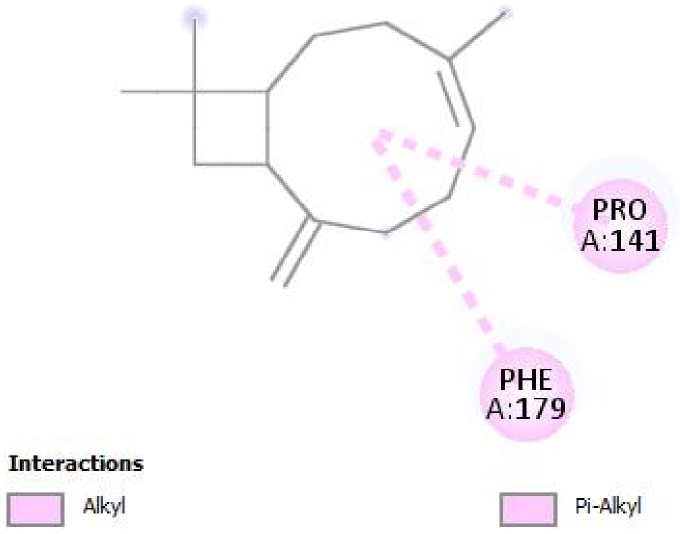
PRO
A:141
PHE
A:179
Interactions
Alkyl
Pi-Alkyl

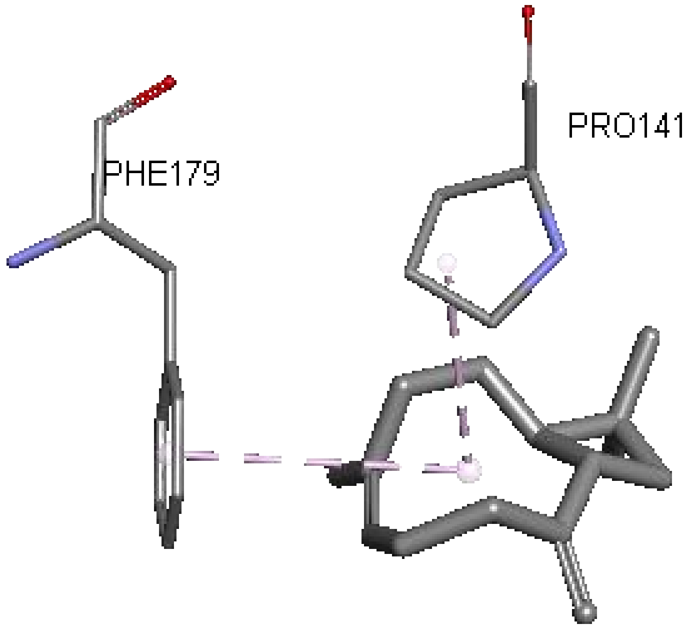
PHE179
PRO141

Protein	1-methyl-4-(propan-2-ylidene)cyclohex-1-ene	
	**2D**	**3D**
1oag	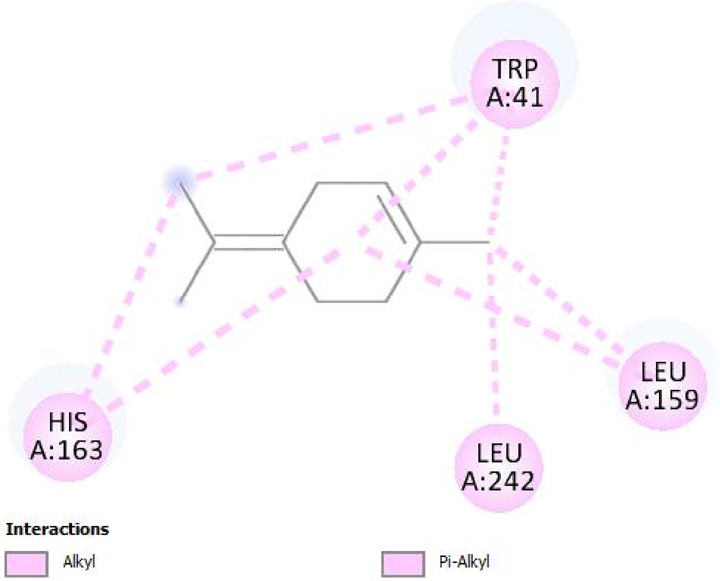	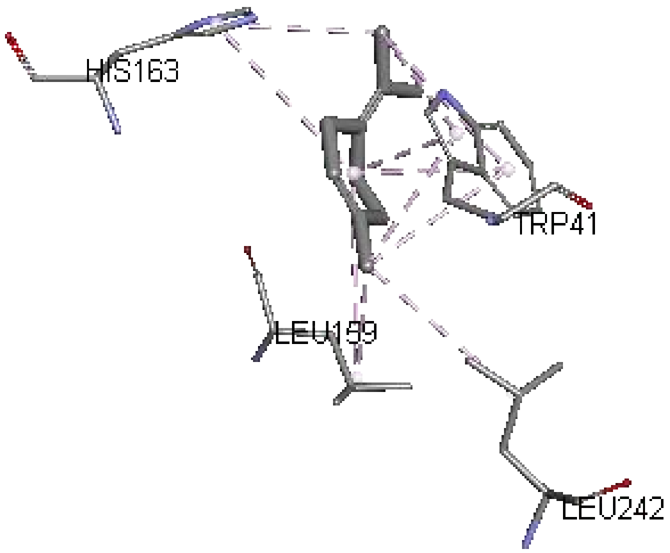

6xv4

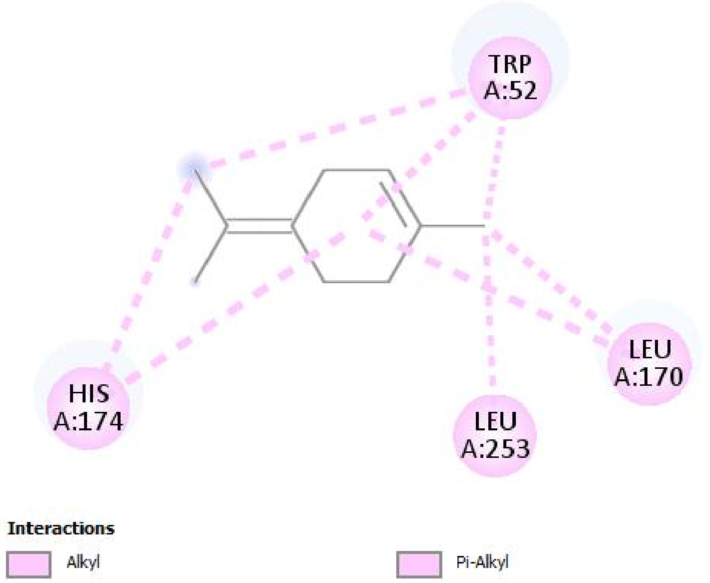

TRP A:52
HIS A:174
LEU A:253
LEU A:170
Interactions
Alkyl
Pi-Alkyl

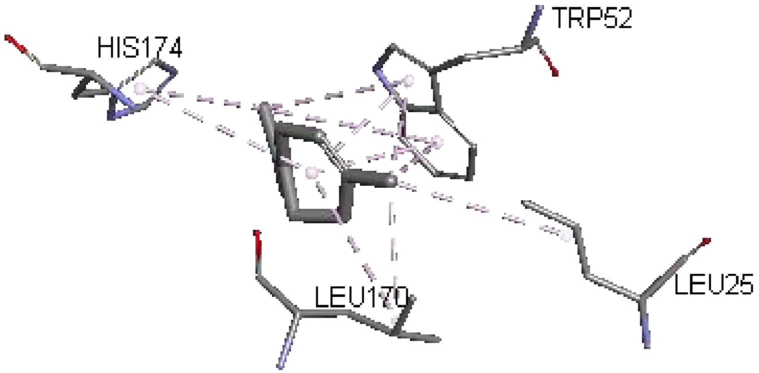

TRP52
HIS174
LEU170
LEU25

9h1m	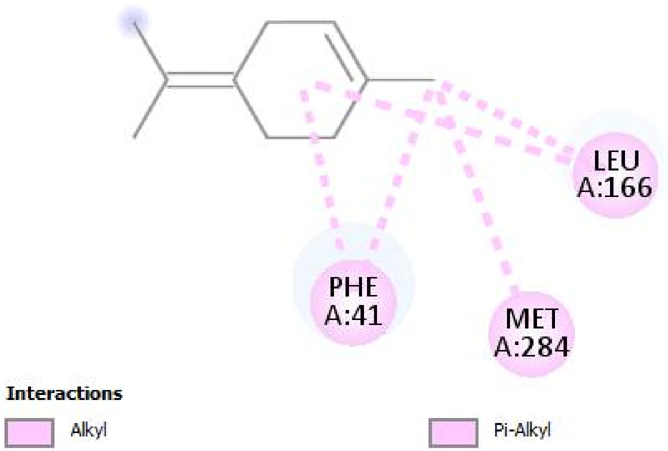LEU A:166
PHE A:41
MET A:284
Interactions
Alkyl
Pi-Alkyl	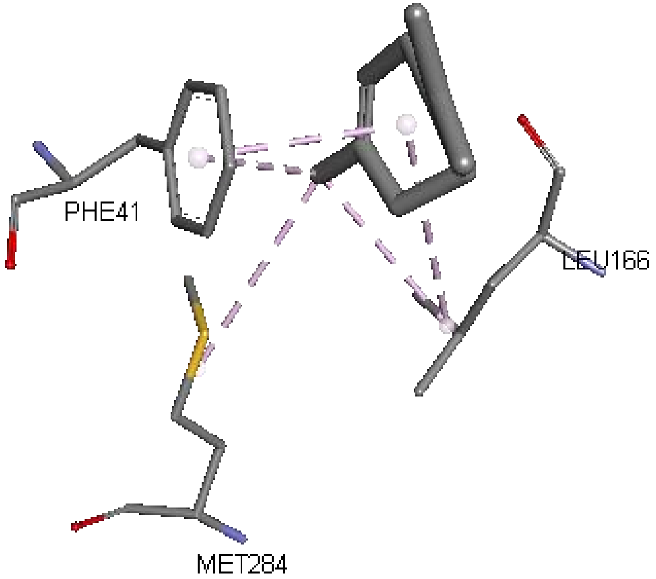PHE41
LEU166
MET284

Protein	1,1,2-trimethyl-3-(2-methylprop-1-en-1-ylidene)cyclopropane	
	**2D**	**3D**
1oag	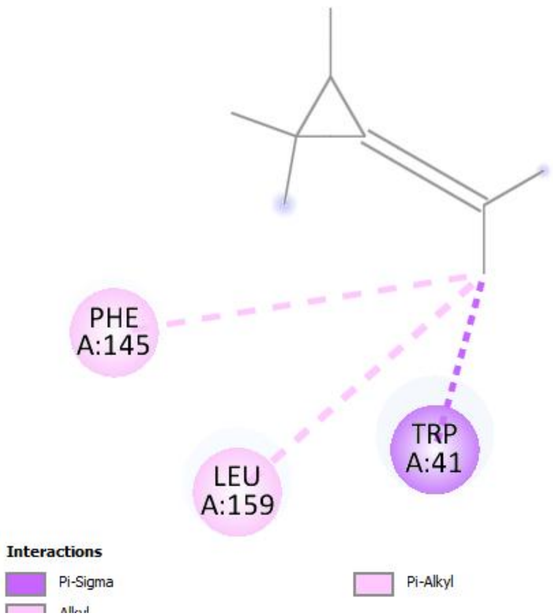	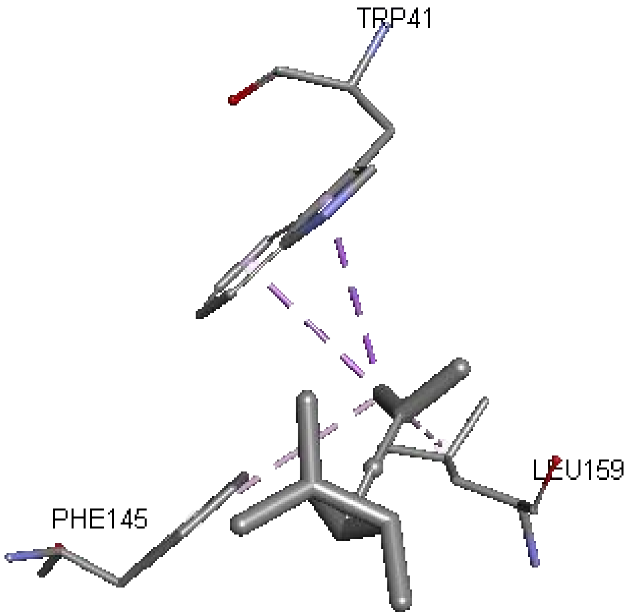

6xv4

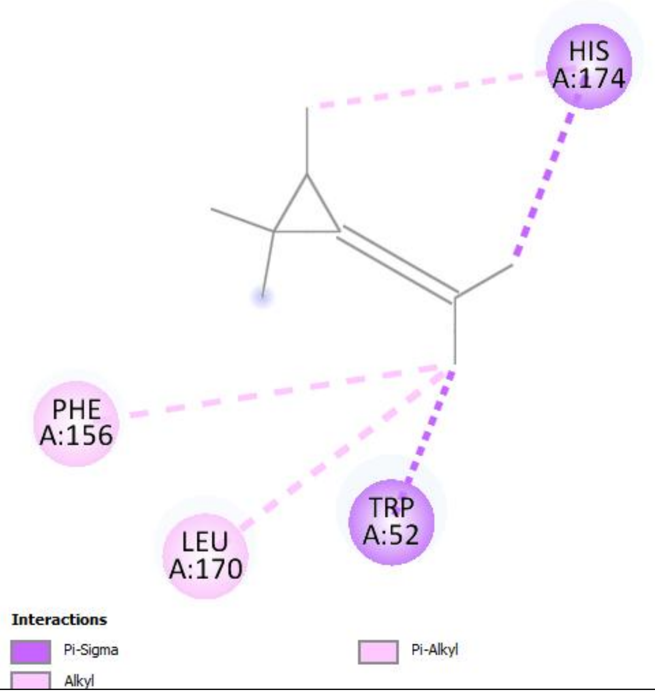

HIS A:174
PHE A:156
LEU A:170
TRP A:52
Interactions
Pi-Sigma
Alkyl
Pi-Alkyl

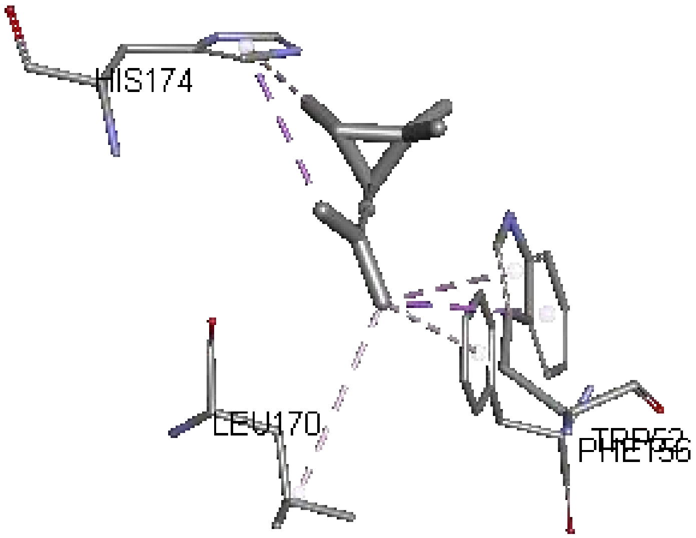

HIS174
LEU170
TRP52
PHE156

9h1m

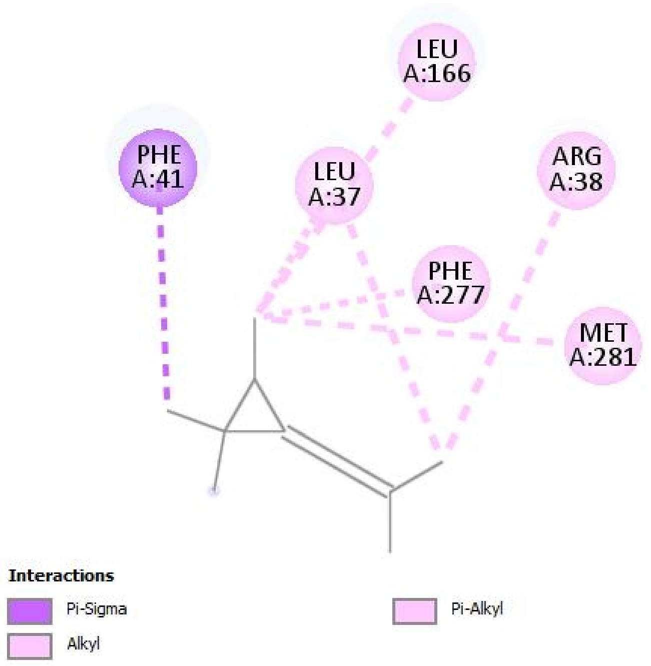

LEU A:166
PHE A:41
LEU A:37
ARG A:38
PHE A:277
MET A:281
Interactions
Pi-Sigma
Alkyl
Pi-Alkyl

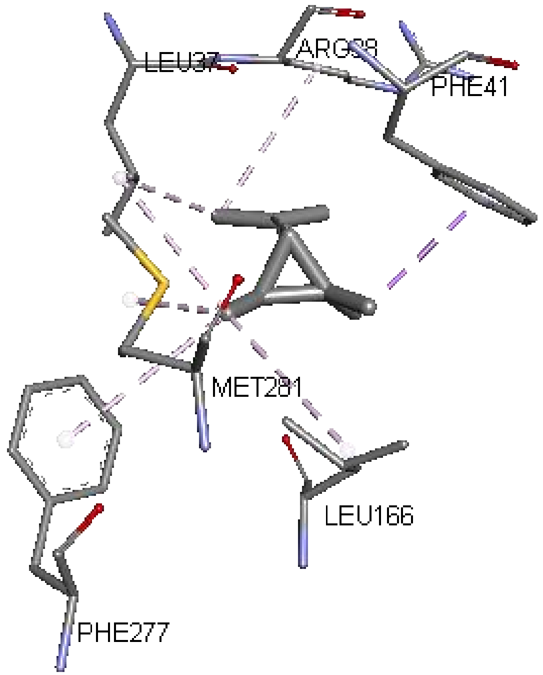

LEU37
PHE41
MET281
LEU166
PHE277

Protein	Protoporphyrin IX containing Fe, co-crystalized ligand (L0)	
	**2D**	**3D**
1oag	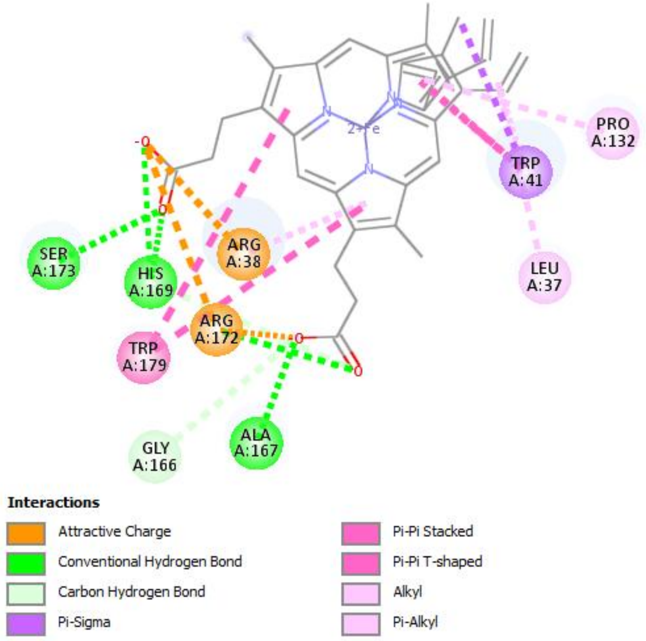	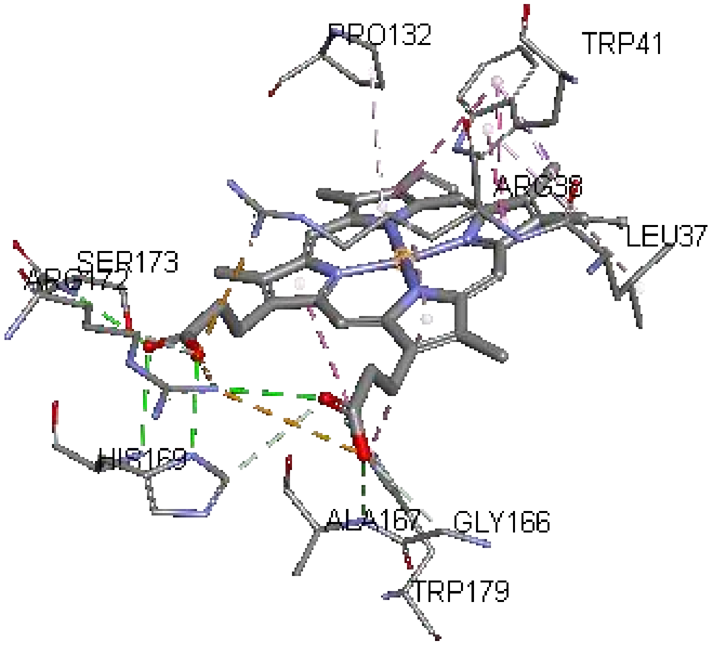

6xv4	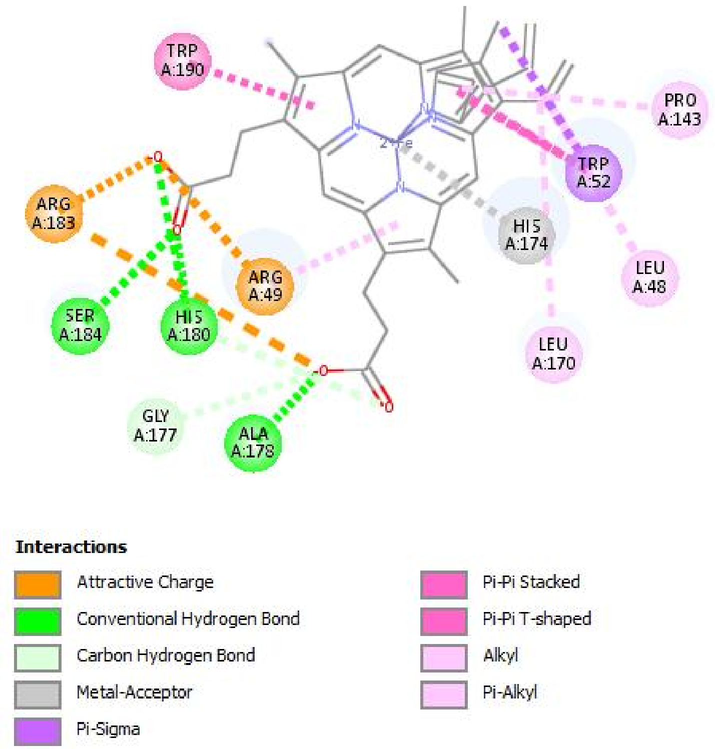	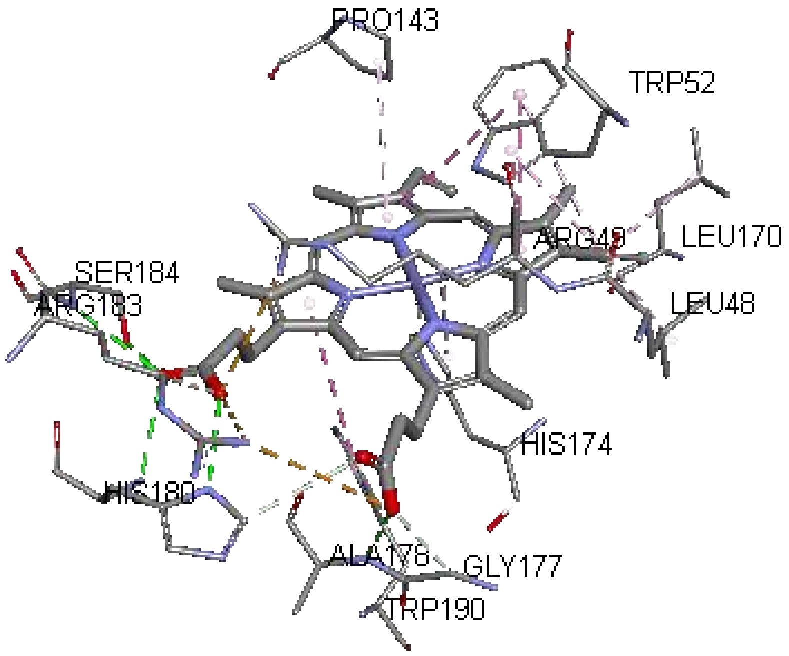

TRP A:190
PRO A:143
TRP A:52
ARG A:183
HIS A:174
ARG A:49
SER A:184
HIS A:180
LEU A:48
LEU A:170
GLY A:177
ALA A:178
Interactions
Attractive Charge
Conventional Hydrogen Bond
Carbon Hydrogen Bond
Metal-Acceptor
Pi-Sigma
Pi-Pi Stacked
Pi-Pi T-shaped
Alkyl
Pi-Alkyl

PRO143
TRP52
LEU170
SER184
ARG183
LEU48
HIS174
ALA178
GLY177
TRP190

9h1m

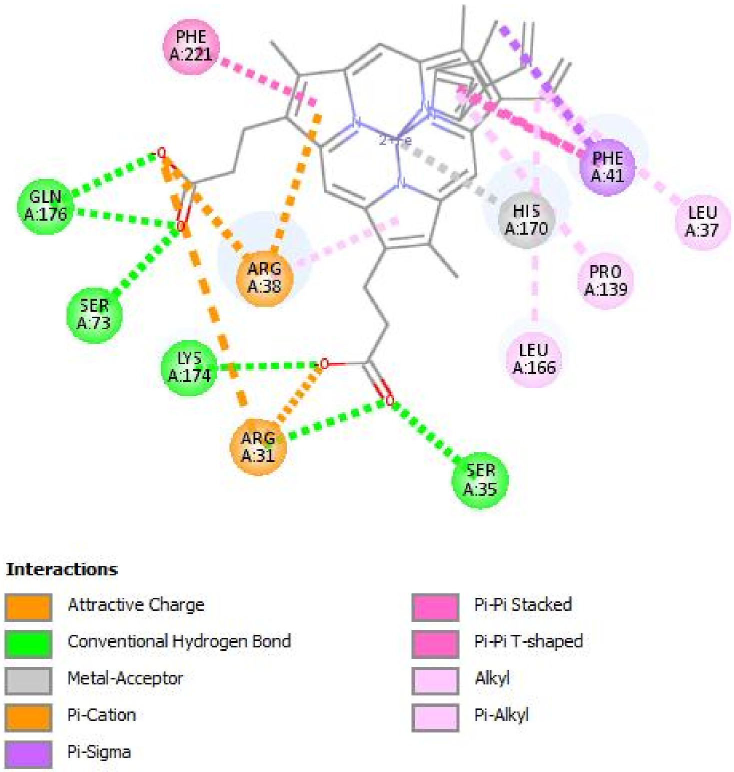
PHE A:221
GLN A:176
SER A:73
LYS A:174
ARG A:31
SER A:35
ARG A:38
HIS A:170
PHE A:41
LEU A:37
PRO A:139
LEU A:166
Interactions
Attractive Charge
Conventional Hydrogen Bond
Metal-Acceptor
Pi-Cation
Pi-Sigma
Pi-Pi Stacked
Pi-Pi T-shaped
Alkyl
Pi-Alkyl

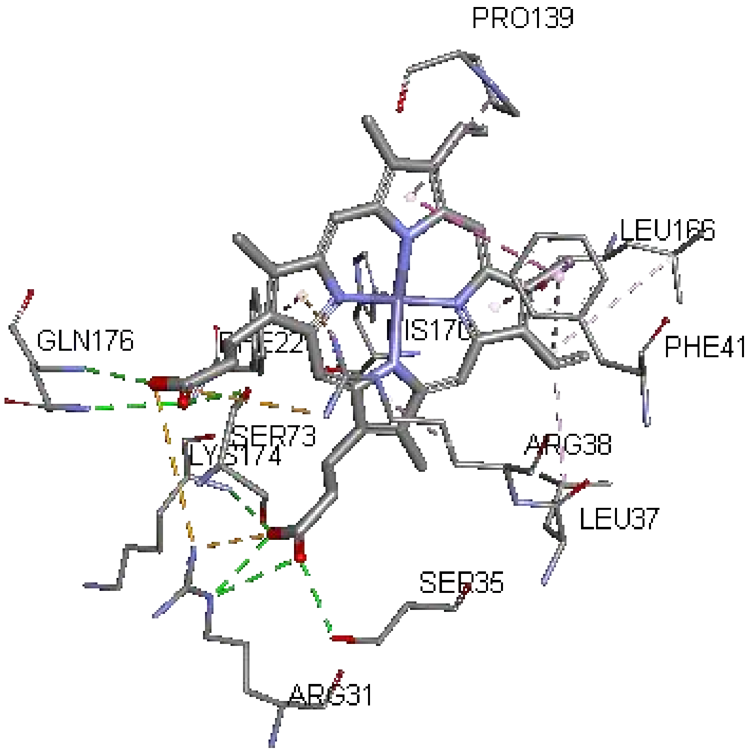
PRO139
LEU166
GLN176
PHE41
SER73
ARG38
LEU37
SER35
ARG31
